# Supplementary material for: Revitalizing Intangible cultural heritage via derivative design: A focus on chinese woodblock printing
Source: PLoS One. 2025 Apr 17;20(4):e0318807. doi: 10.1371/journal.pone.0318807 (PMC12005565; doi:10.1371/journal.pone.0318807)
Supplement: S1 File — (PDF) [file pone.0318807.s001.pdf]

Summary of Design Sketches

Design inspiration diagram

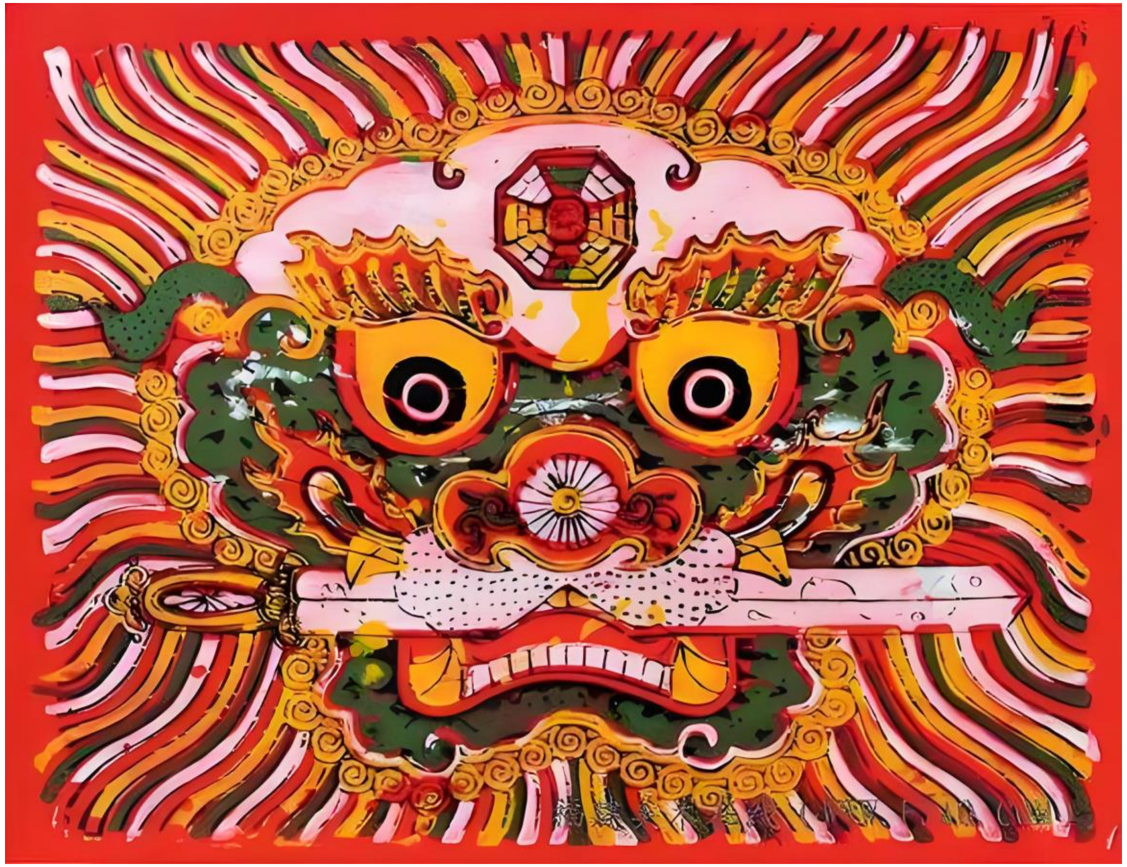

Fig 6. Lion Holding a Sword in Its Mouth（Chinese New Year woodblock prints）

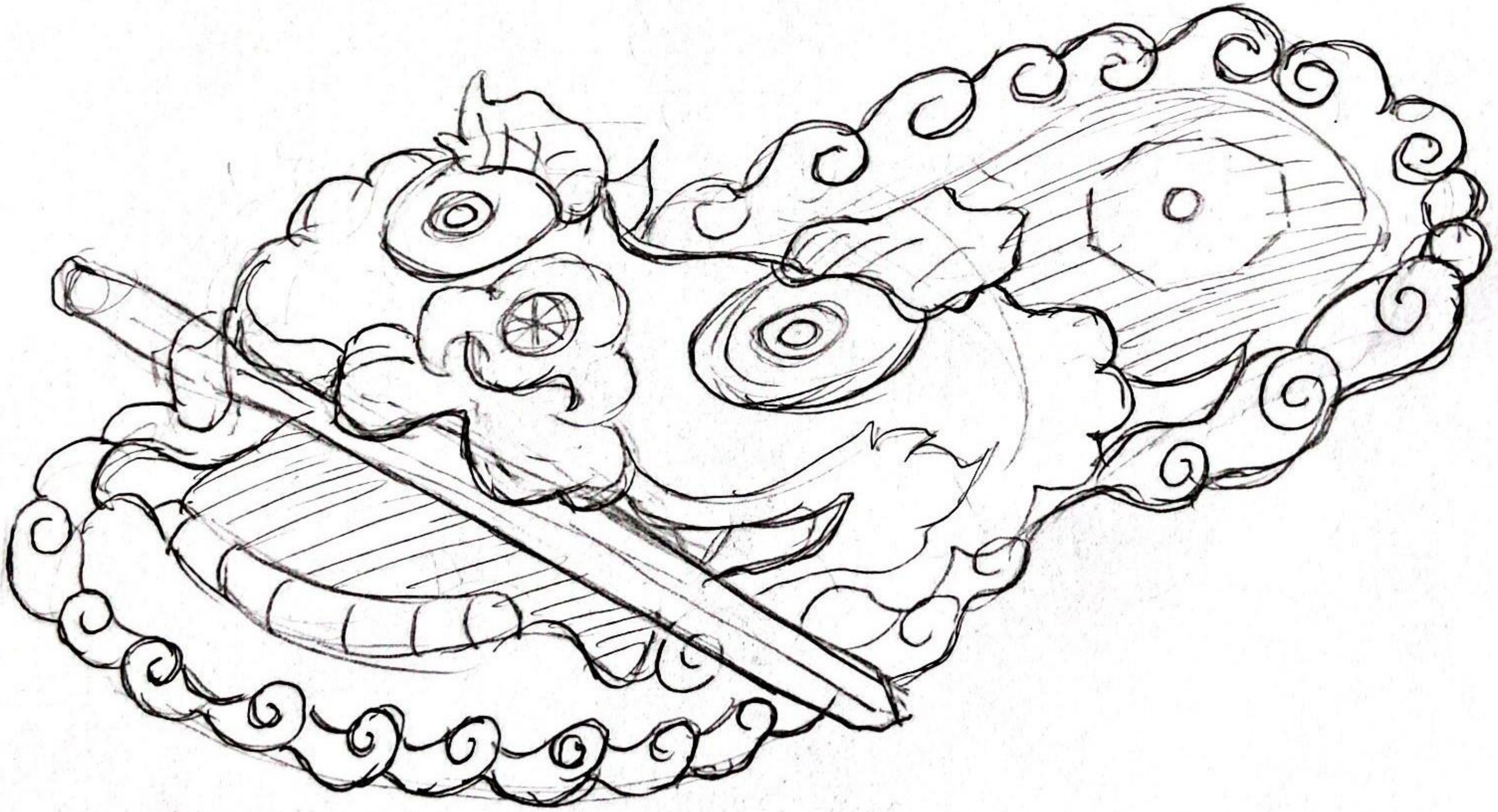

陆冠廷
172002102

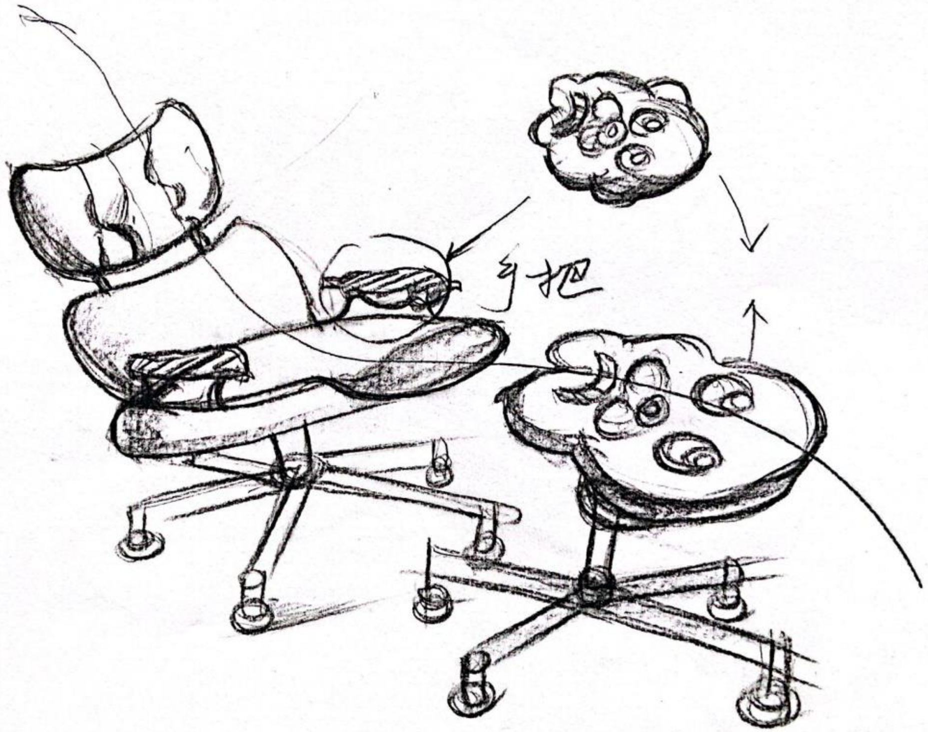

17200252
樊力嘉

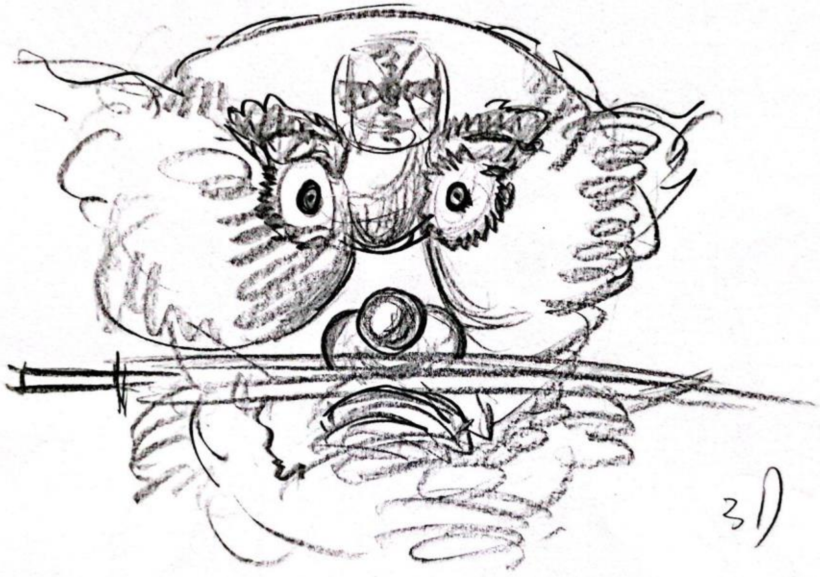

31

[illegible]

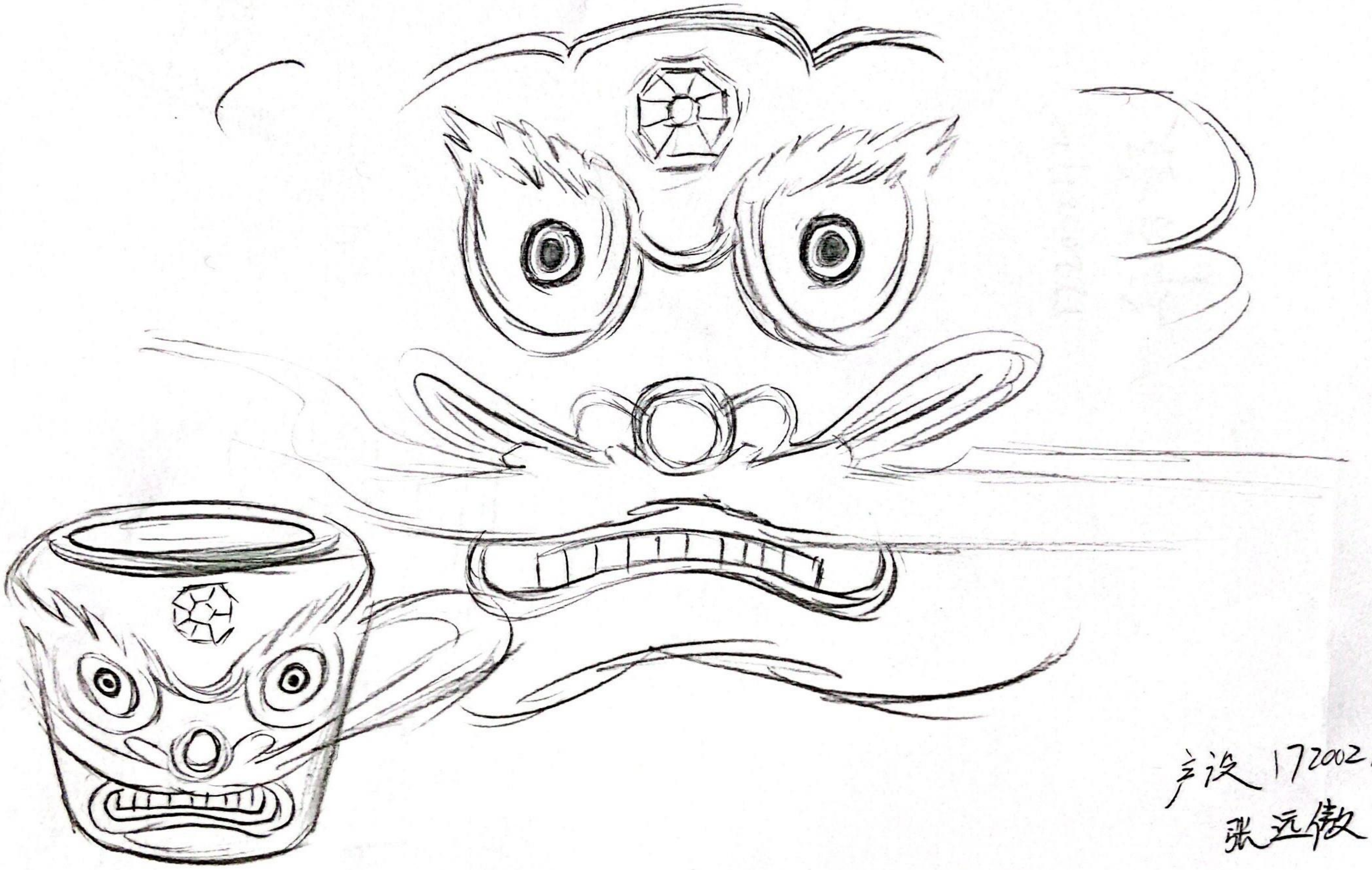

产设 172002106
张远傲

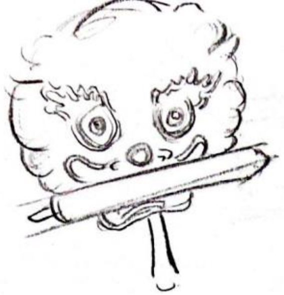

扇子

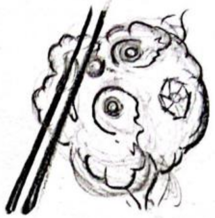

文[illegible]

挂件

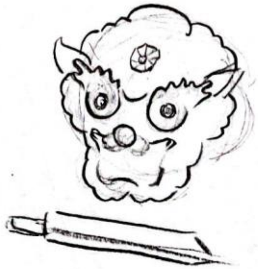

豆画

~~加湿器~~

172002112

郑雨[illegible]

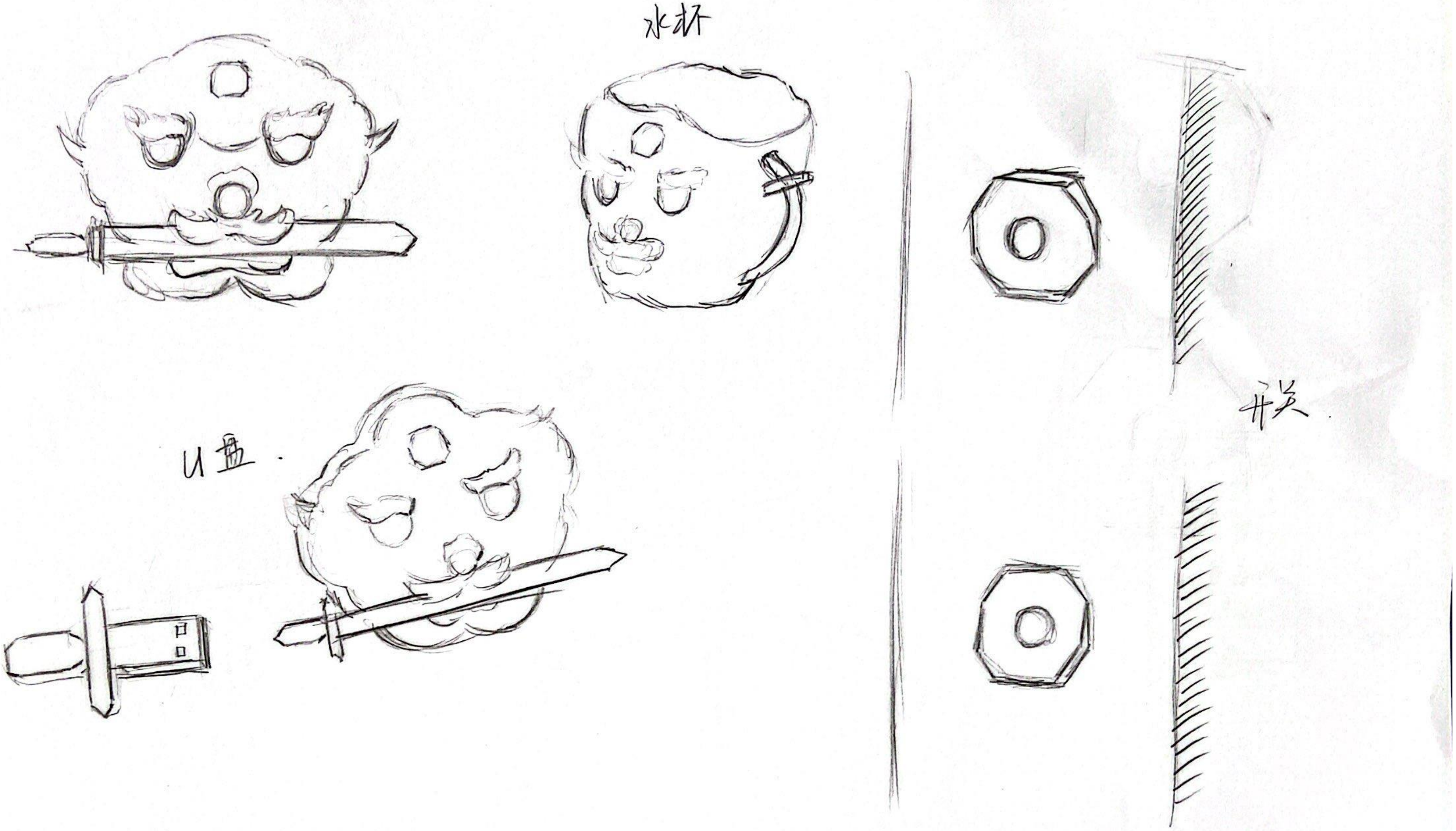

产品一班 172002103 郑婕

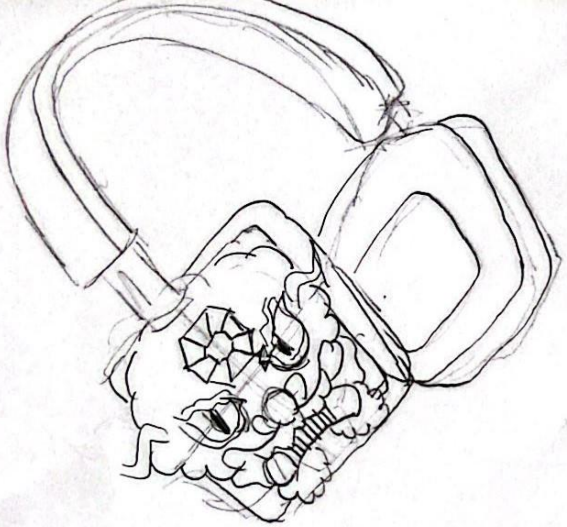

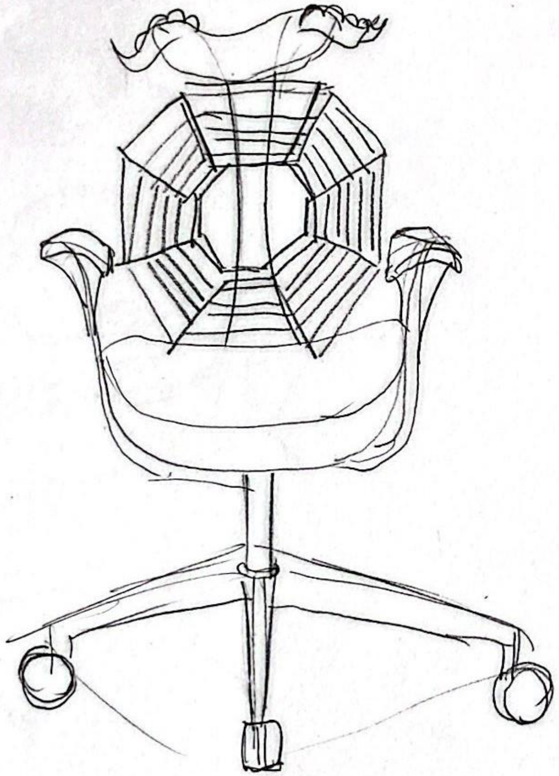

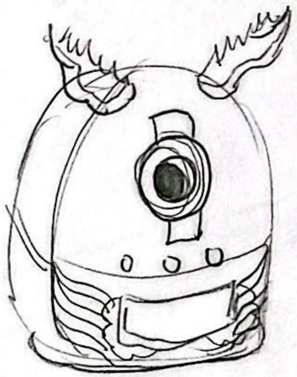

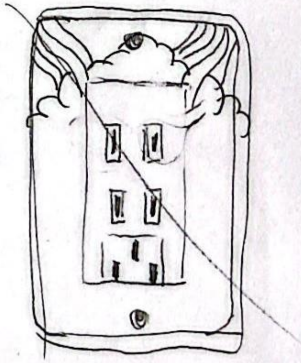

郑[illegible]

产1

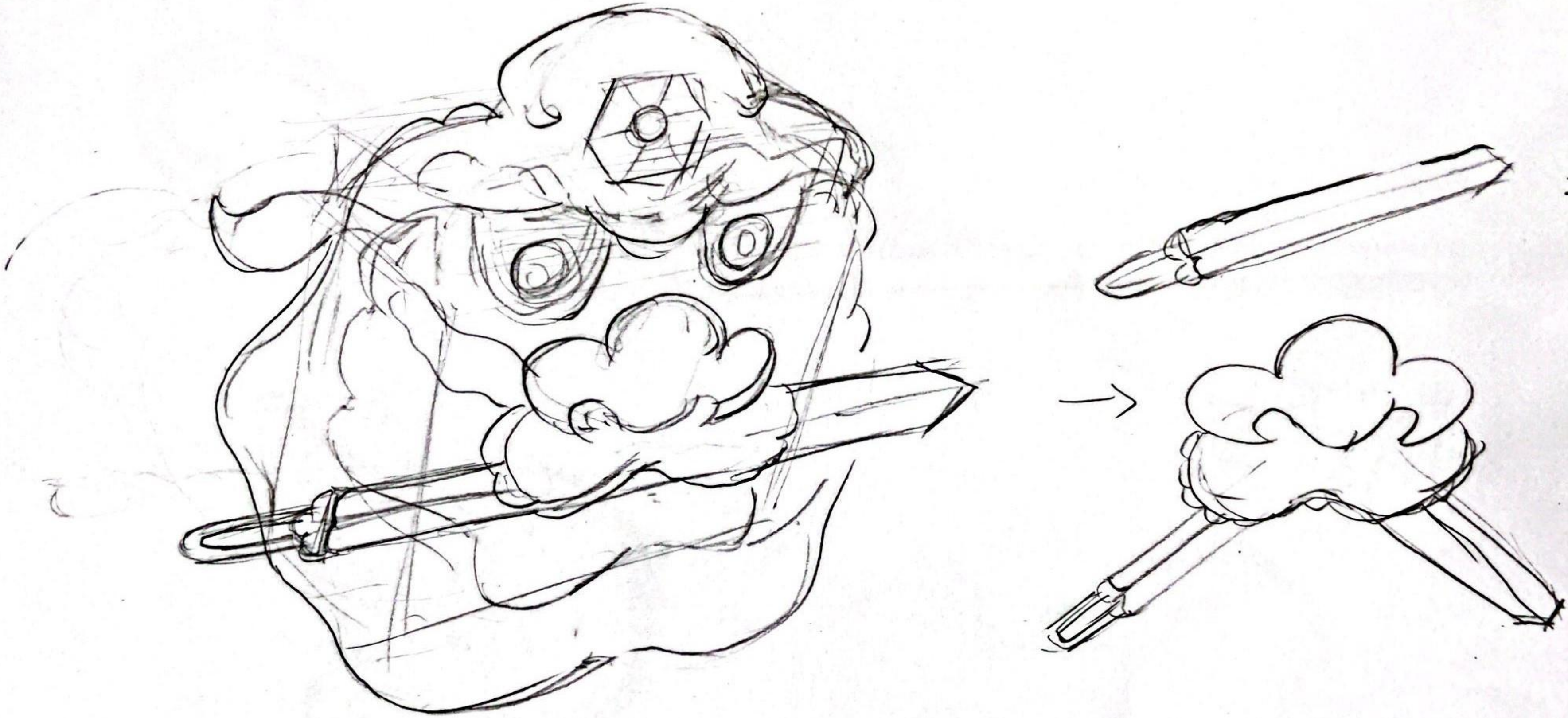

门锁

17200 1033

任东依

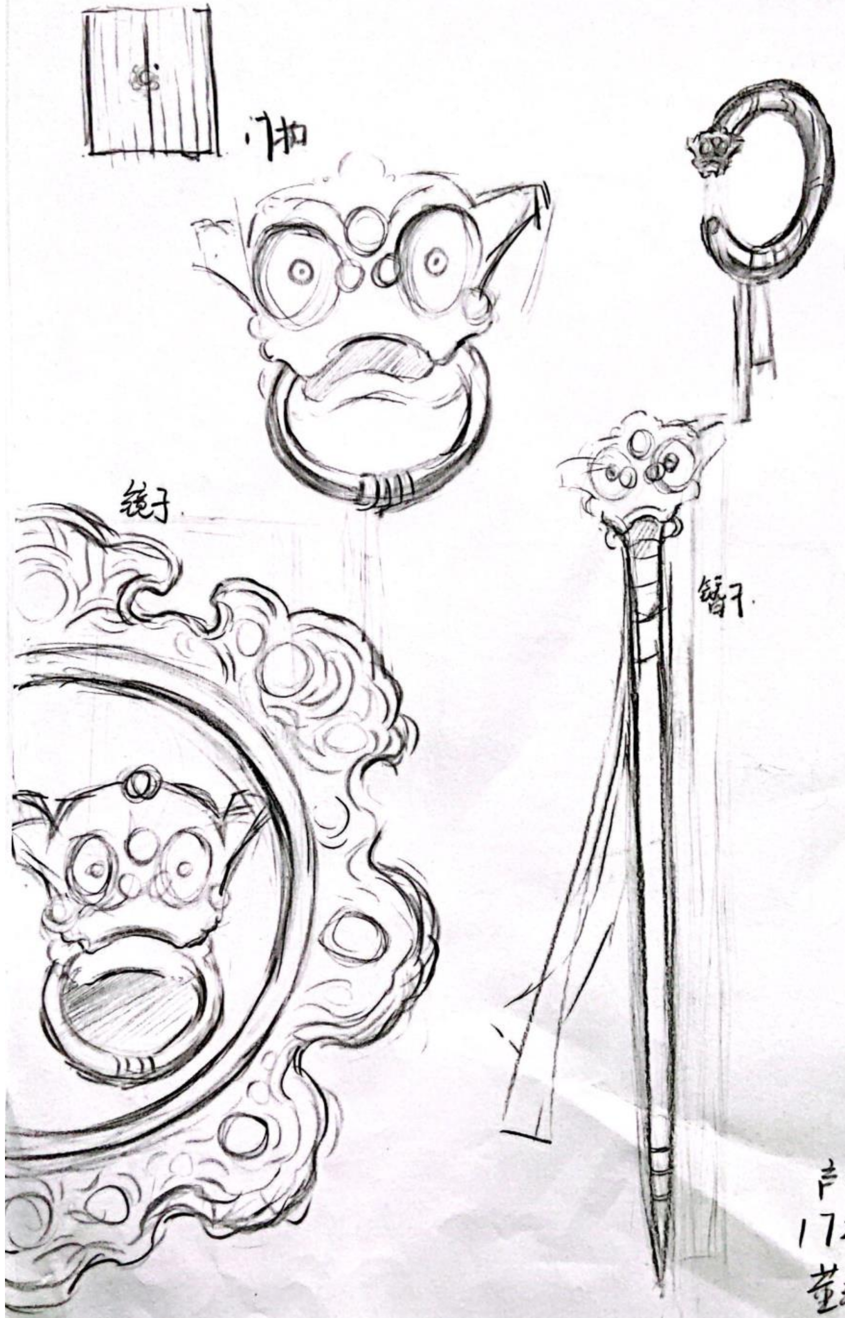

产品一.
1720021192.
董[illegible].

胡晓珍
172002197

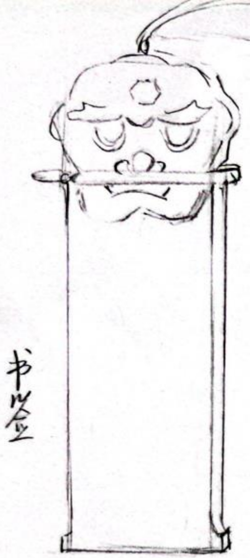

书签

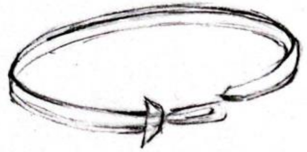

手环

水杯

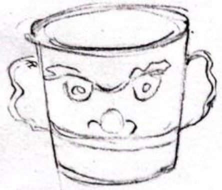

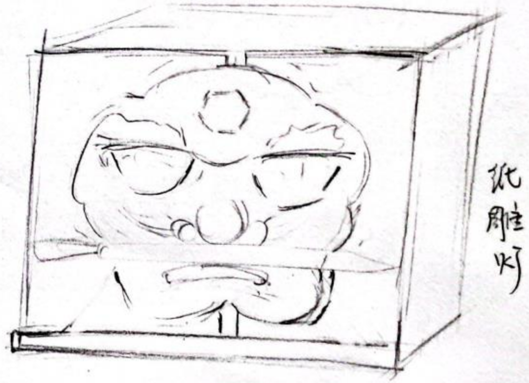

纸雕灯

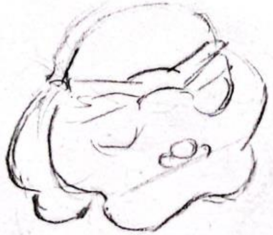

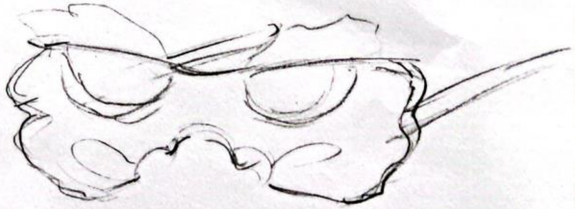

夹子 例

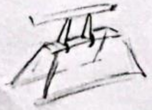

眼镜

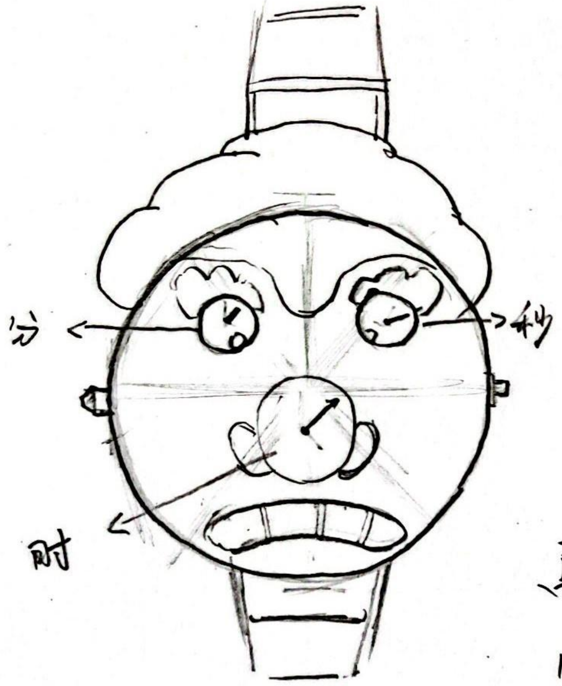

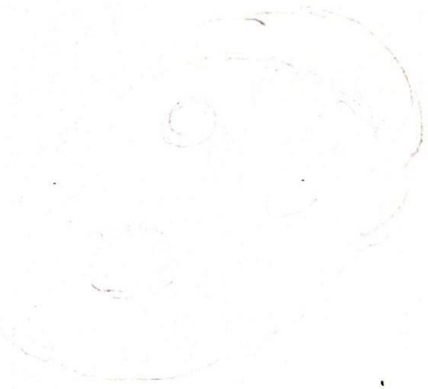

马博扬

172002157

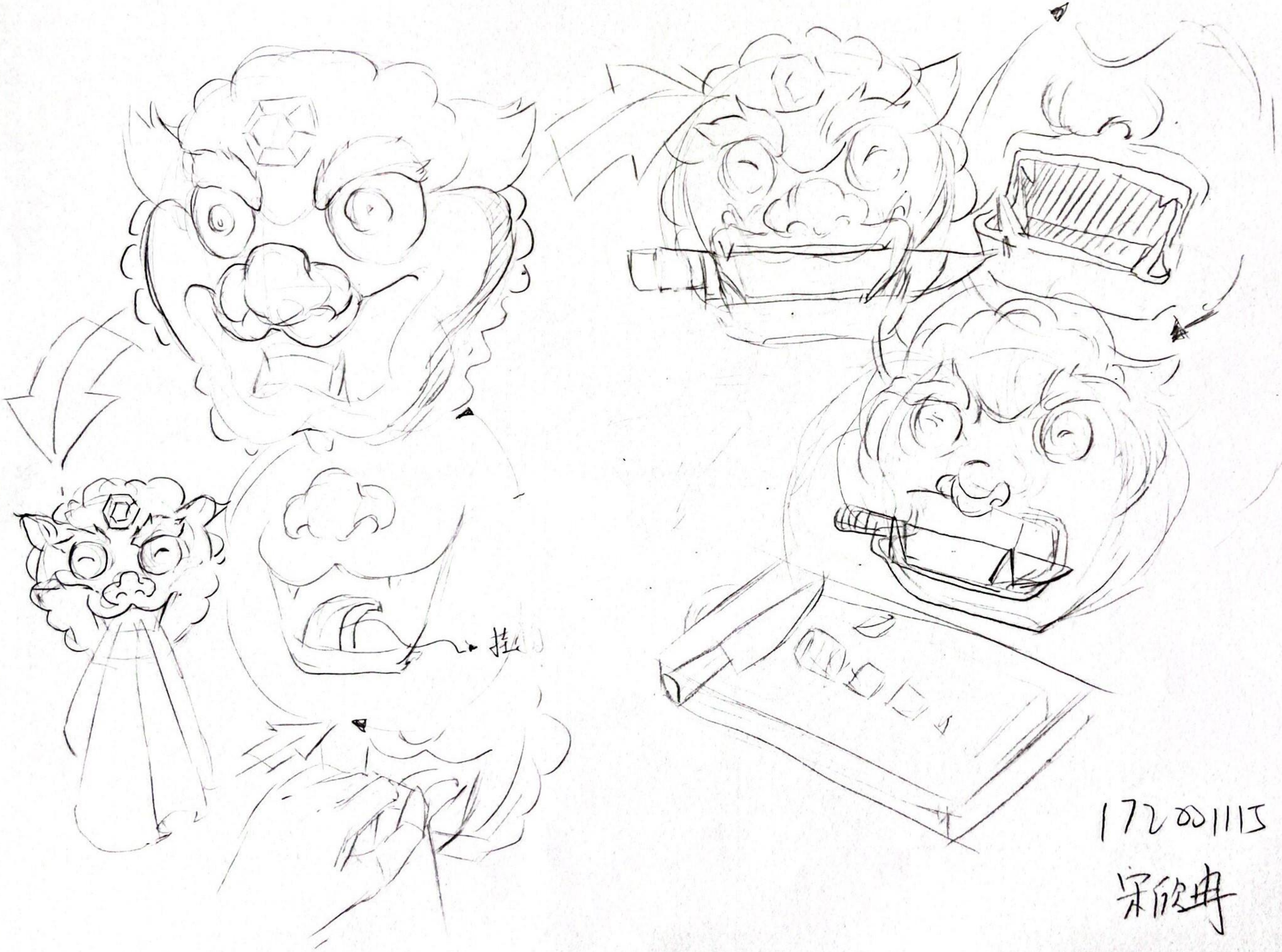

172001115

宋欣冉

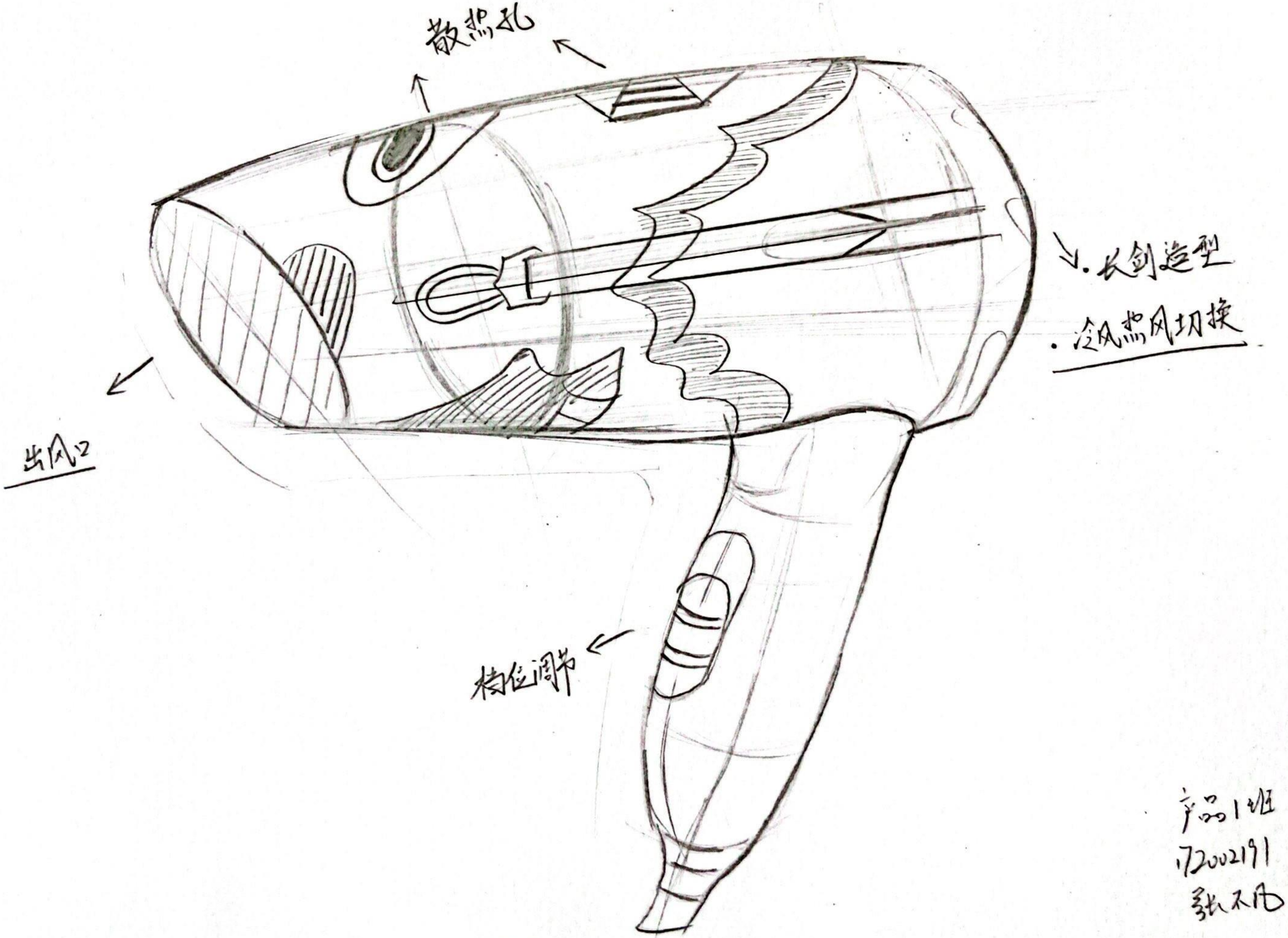

产品1班
172002191
张不凡

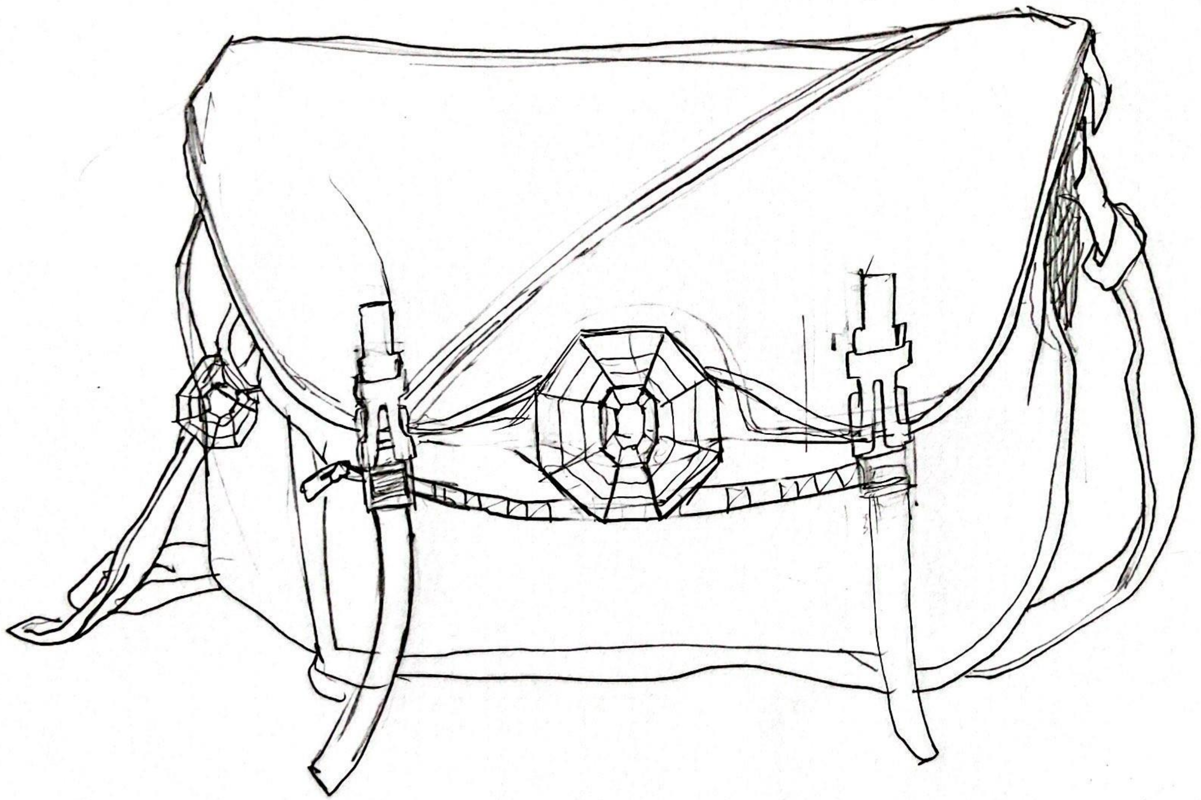

赵昀煋

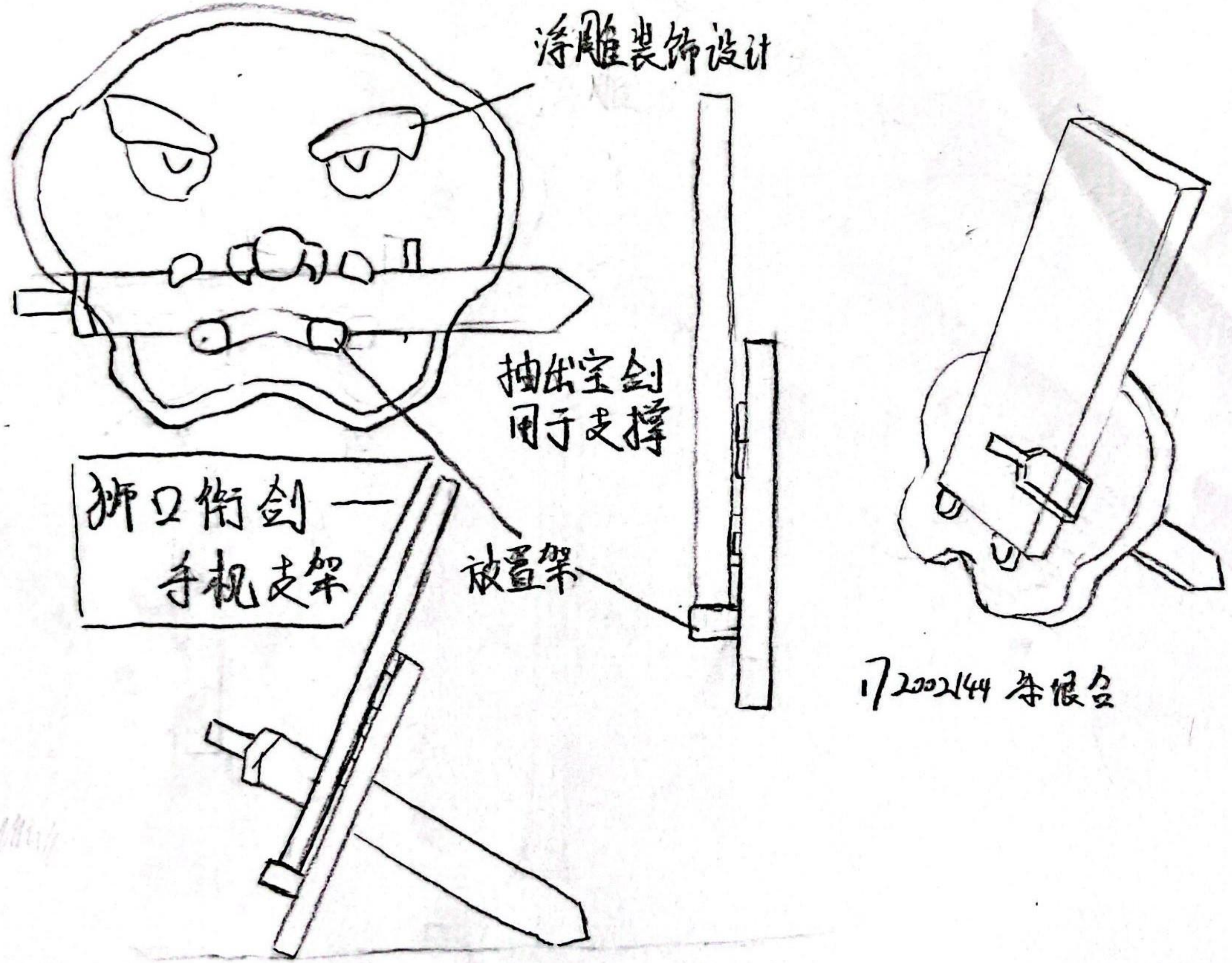

17 2002144 [illegible]

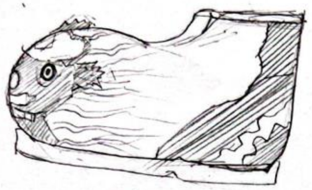

娃娃鞋

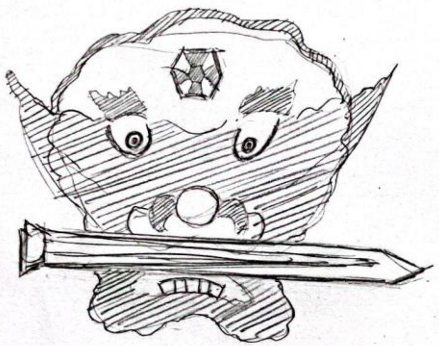

发夹

安子豪
172002175
产品1班

狮子加湿器

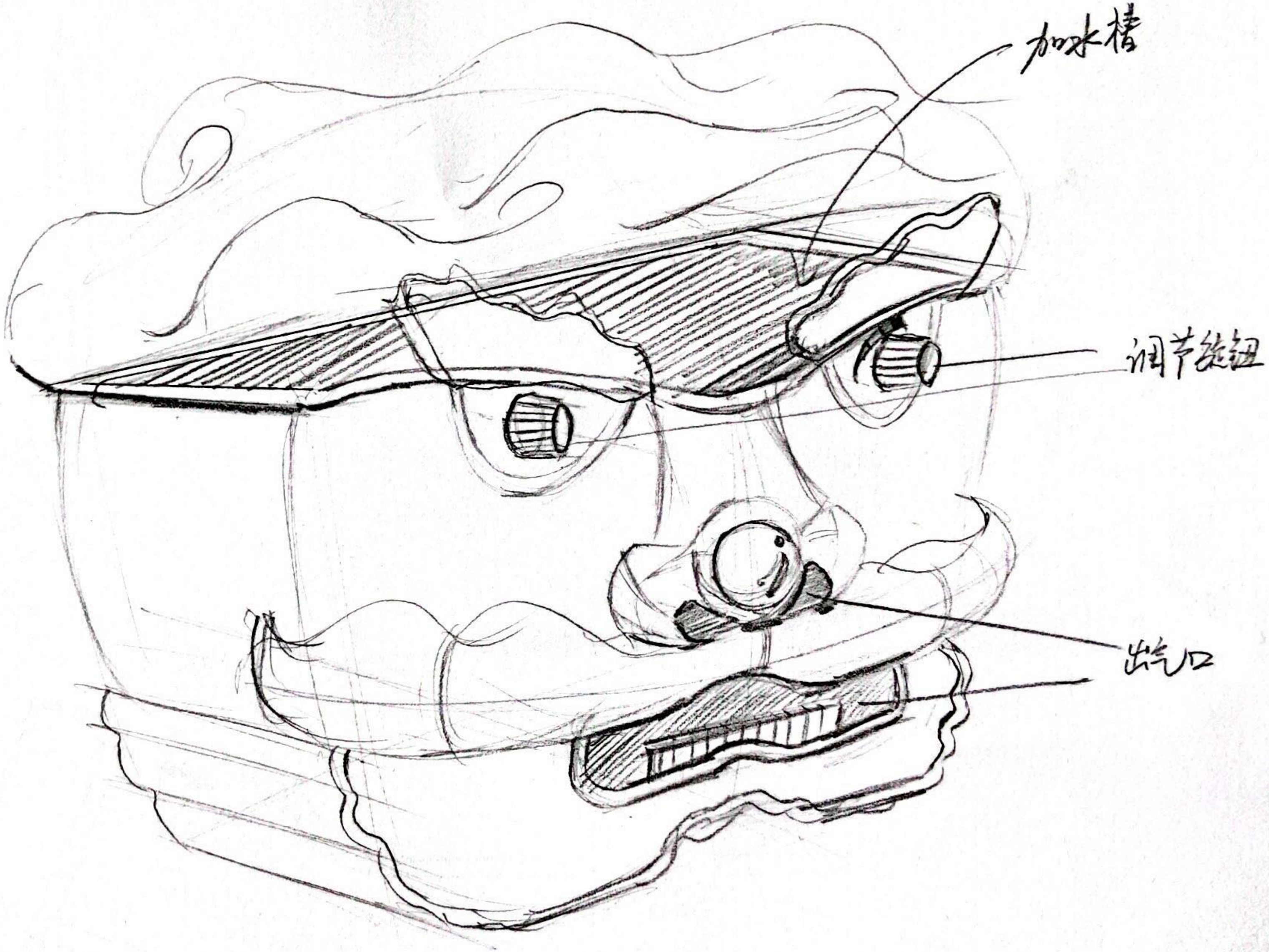

17202261 周[illegible]

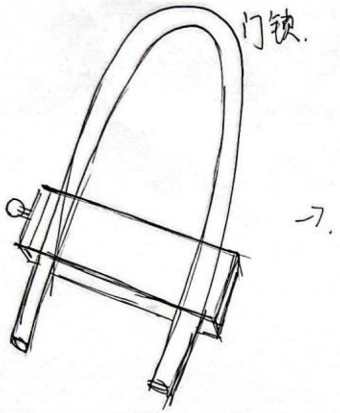

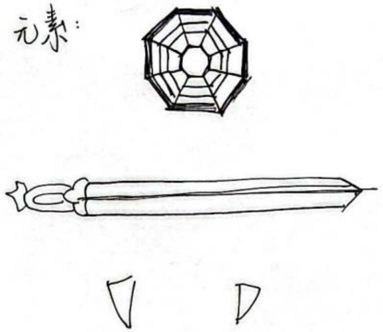

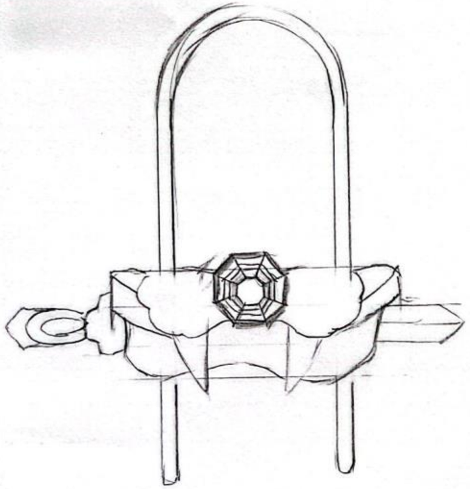

产1 崔文艳
172002056.

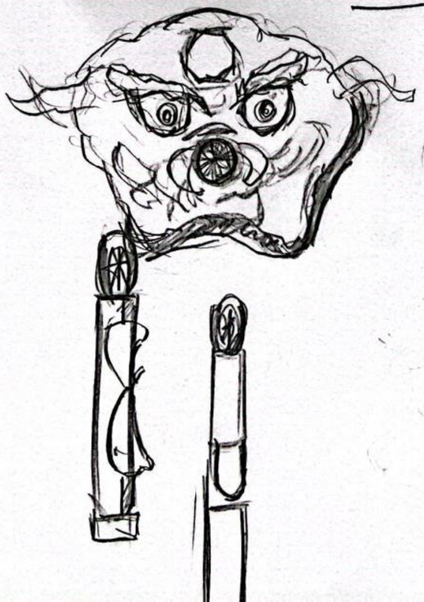

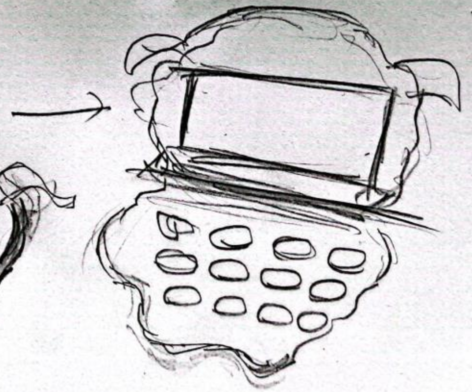

張濬文 112002021

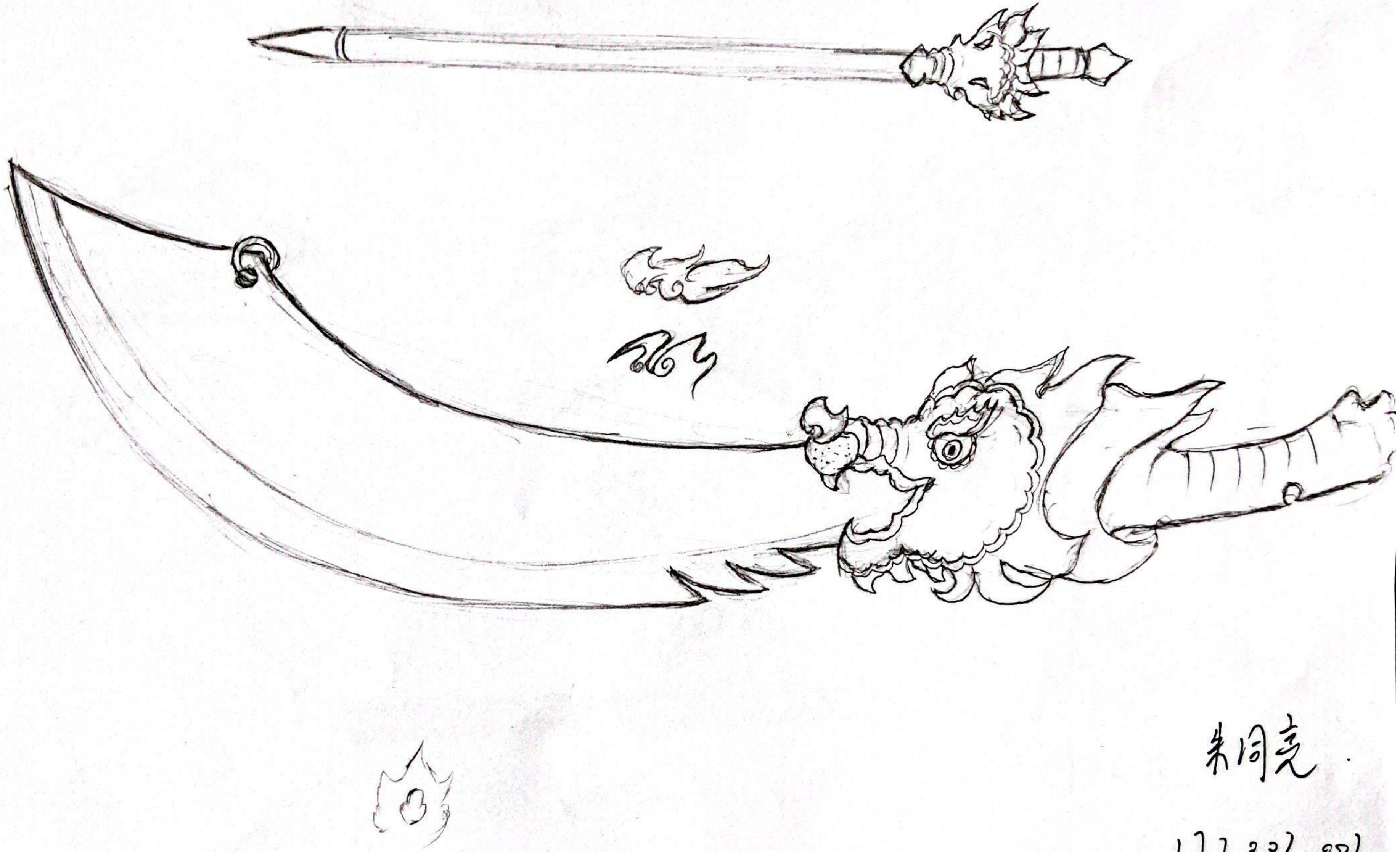

朱同亮.

172002002

172002001 查本出

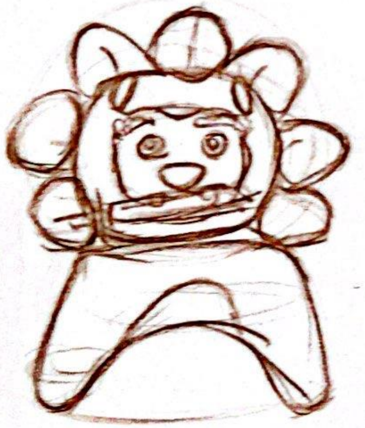

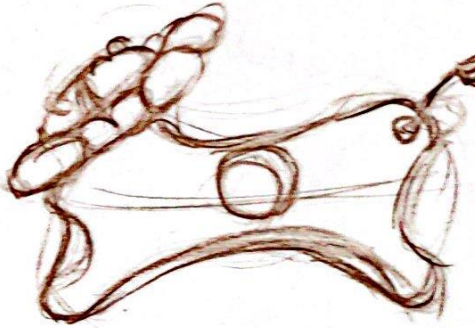

笔架

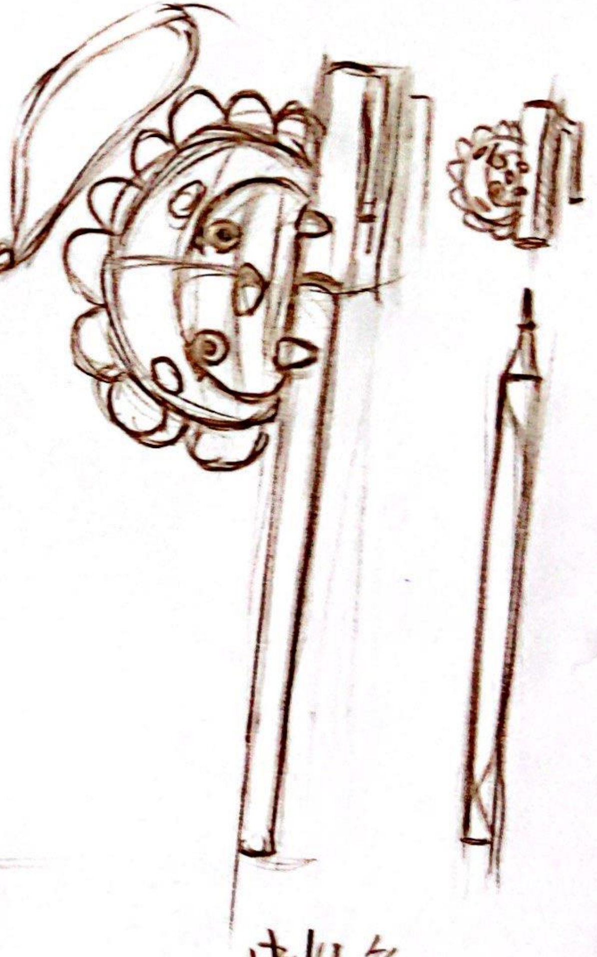

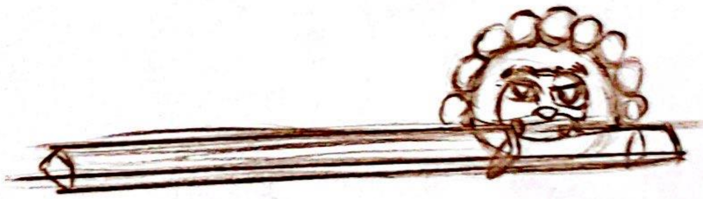

温度计盒

中性笔

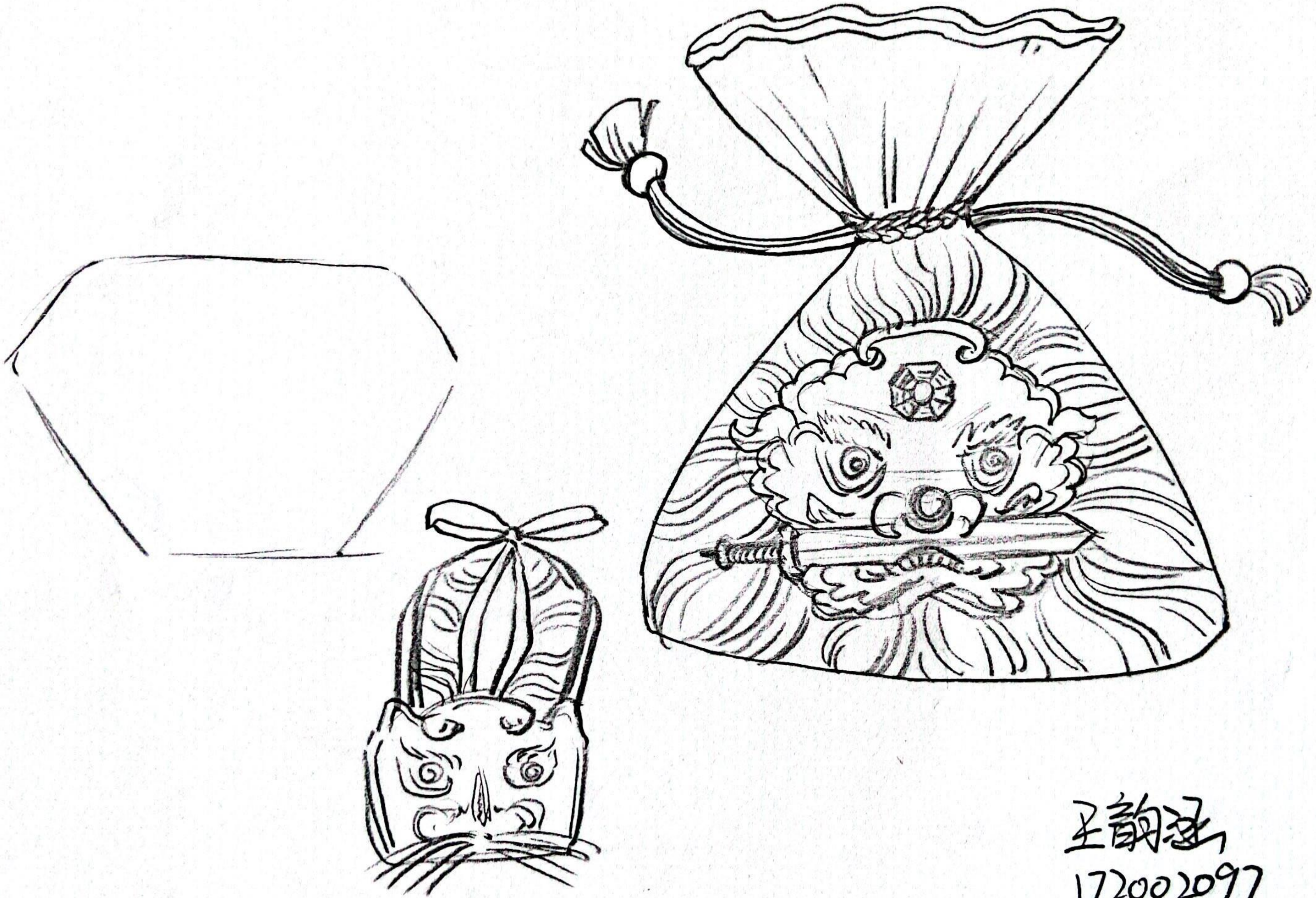

王韵涵
172002097

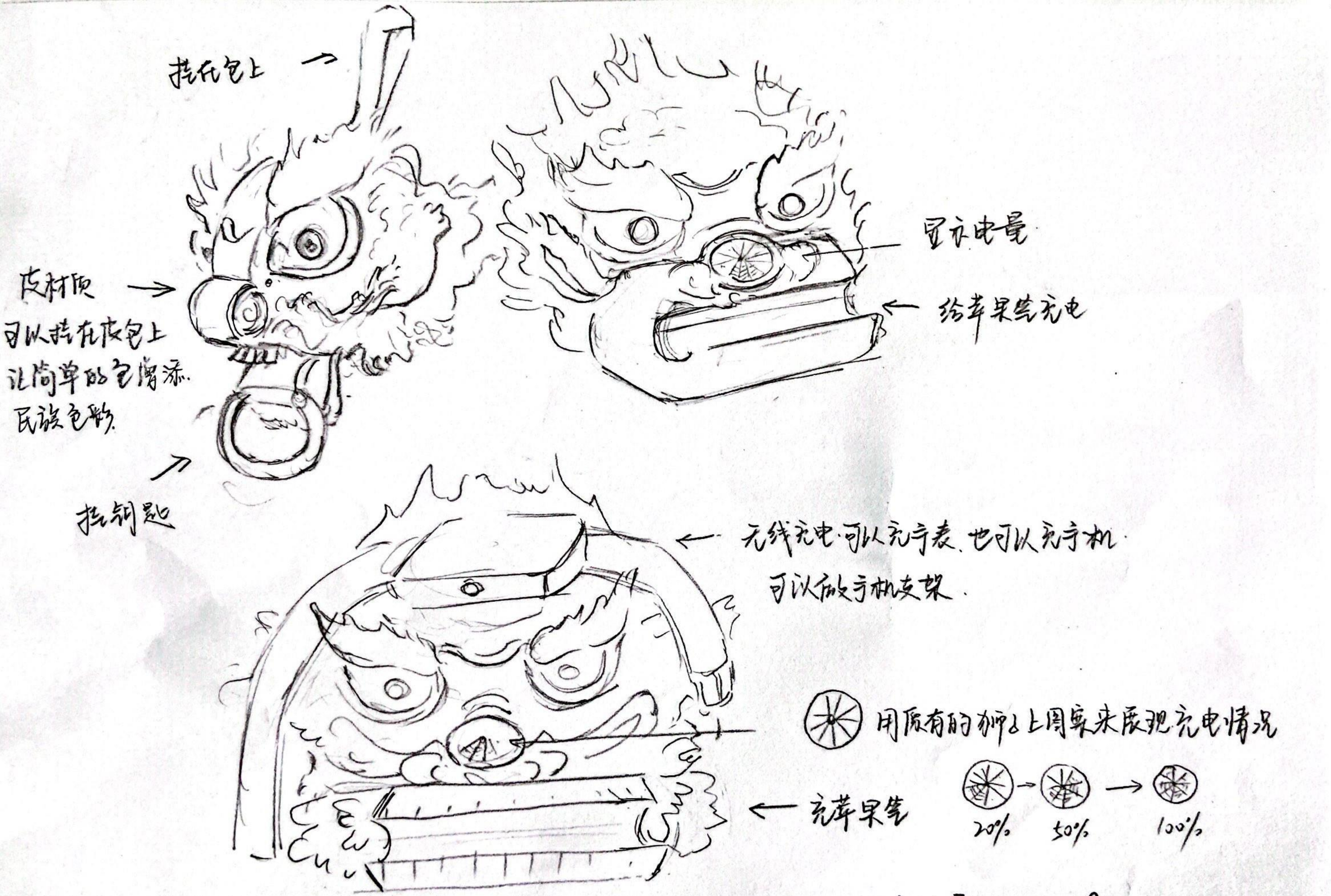

孙诗序. 17203091

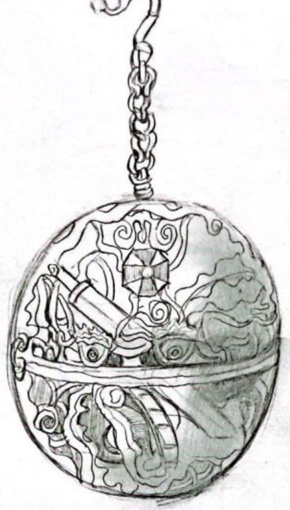

陆嘉玲
17200208

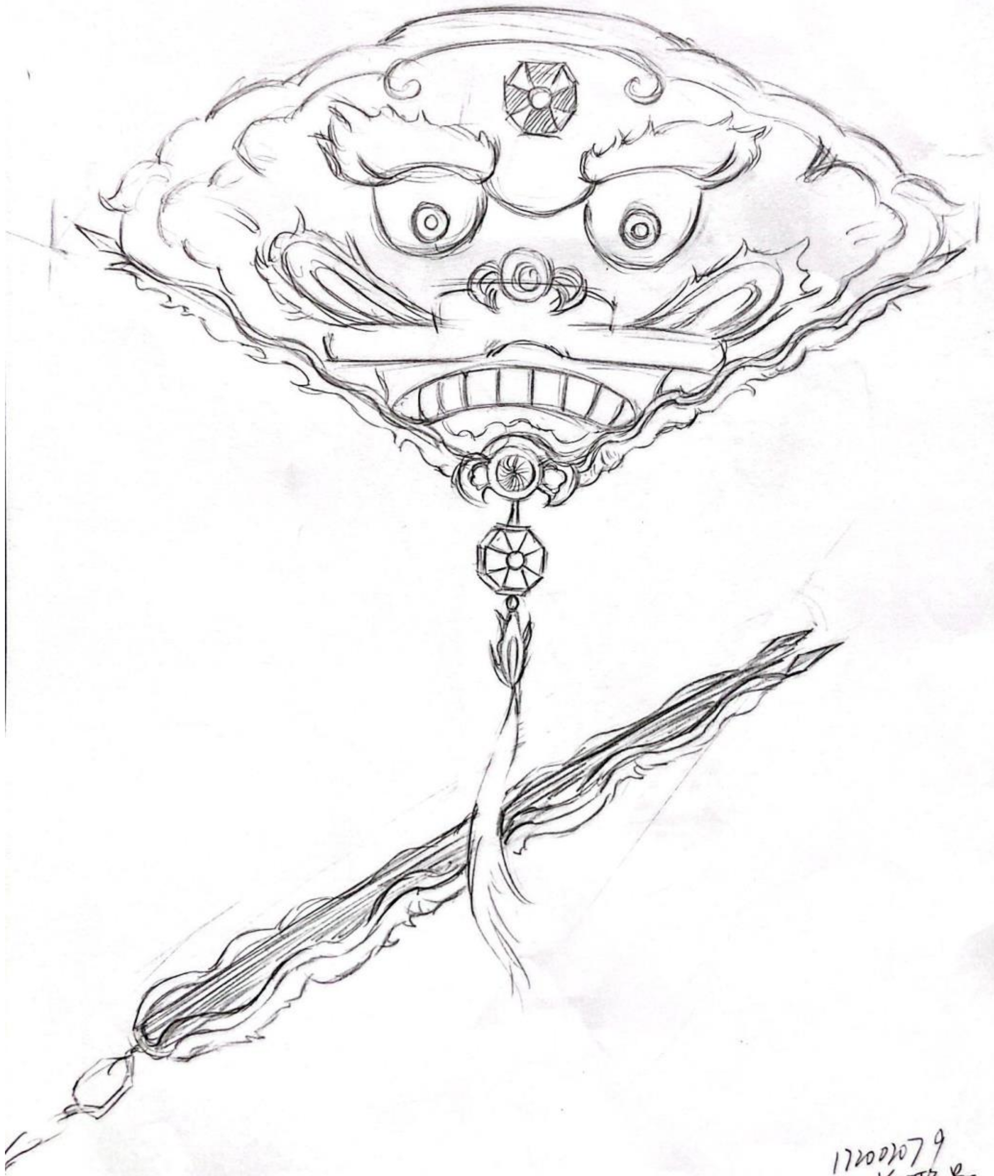

172002079
[illegible]

17200172 王[illegible]武

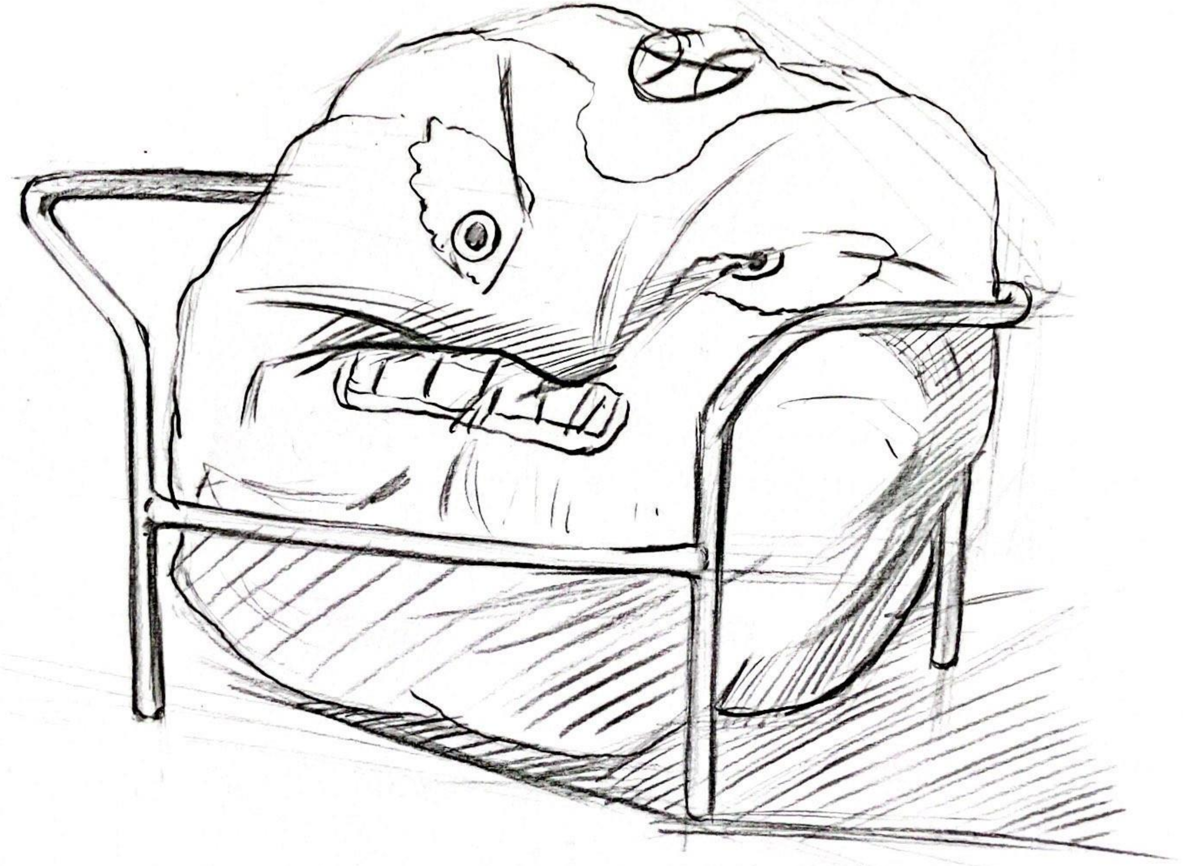

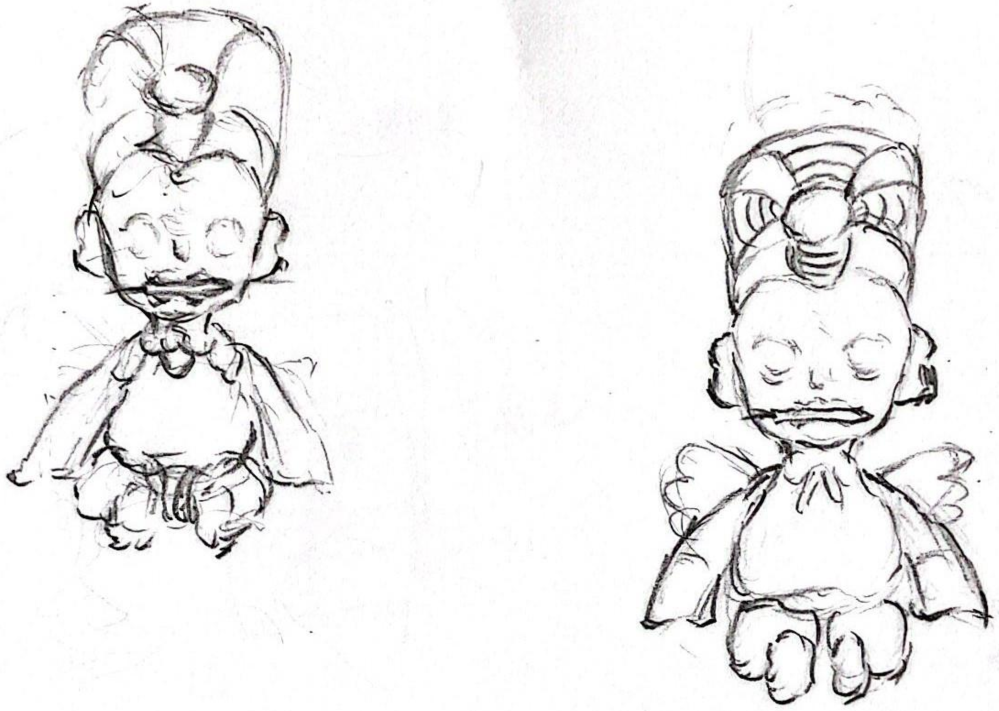

卢佳

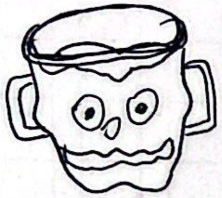

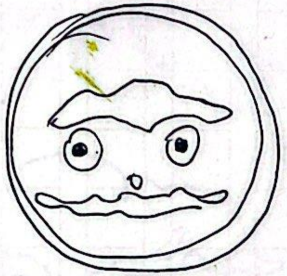

徽章

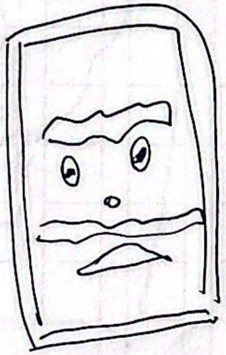

手机壳

张梦雨 172002155

香包.

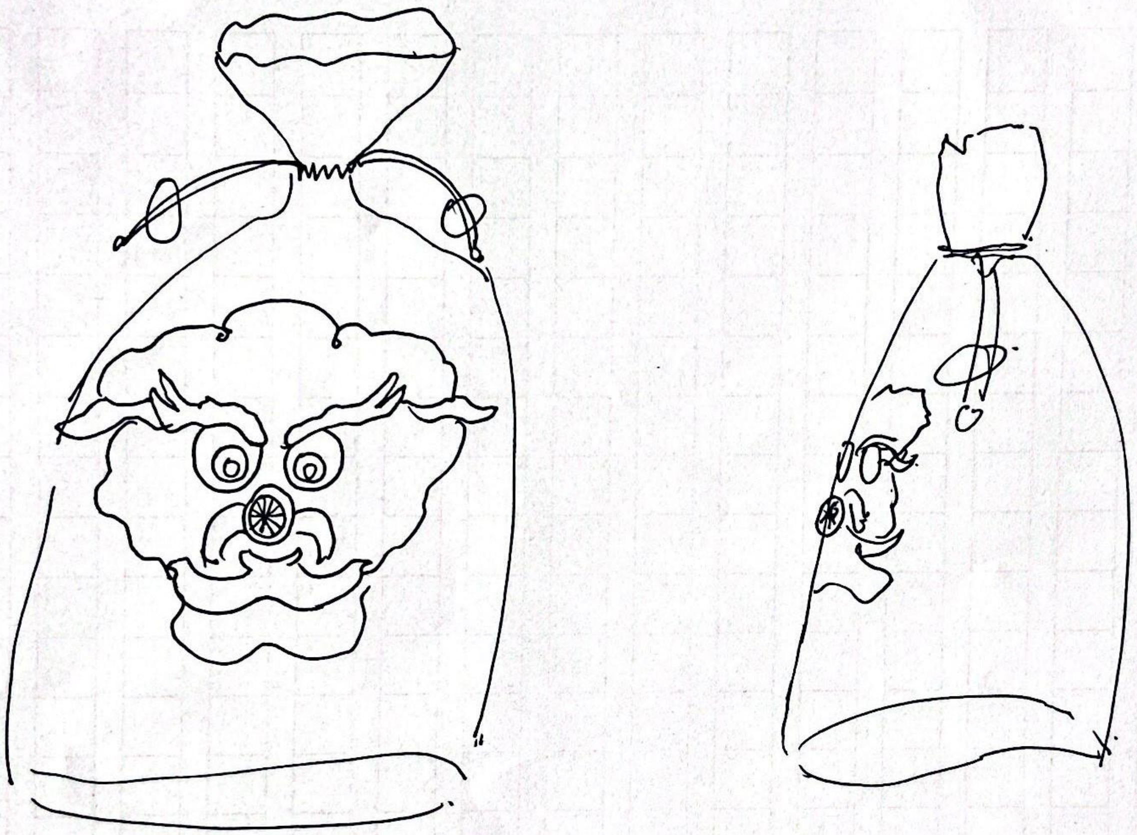

车载挂饰.

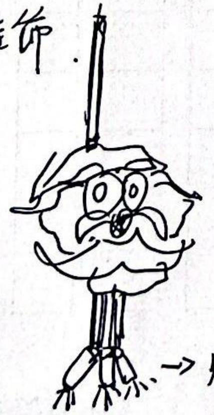

→ 爆竹.

顾信佳.
172002142.

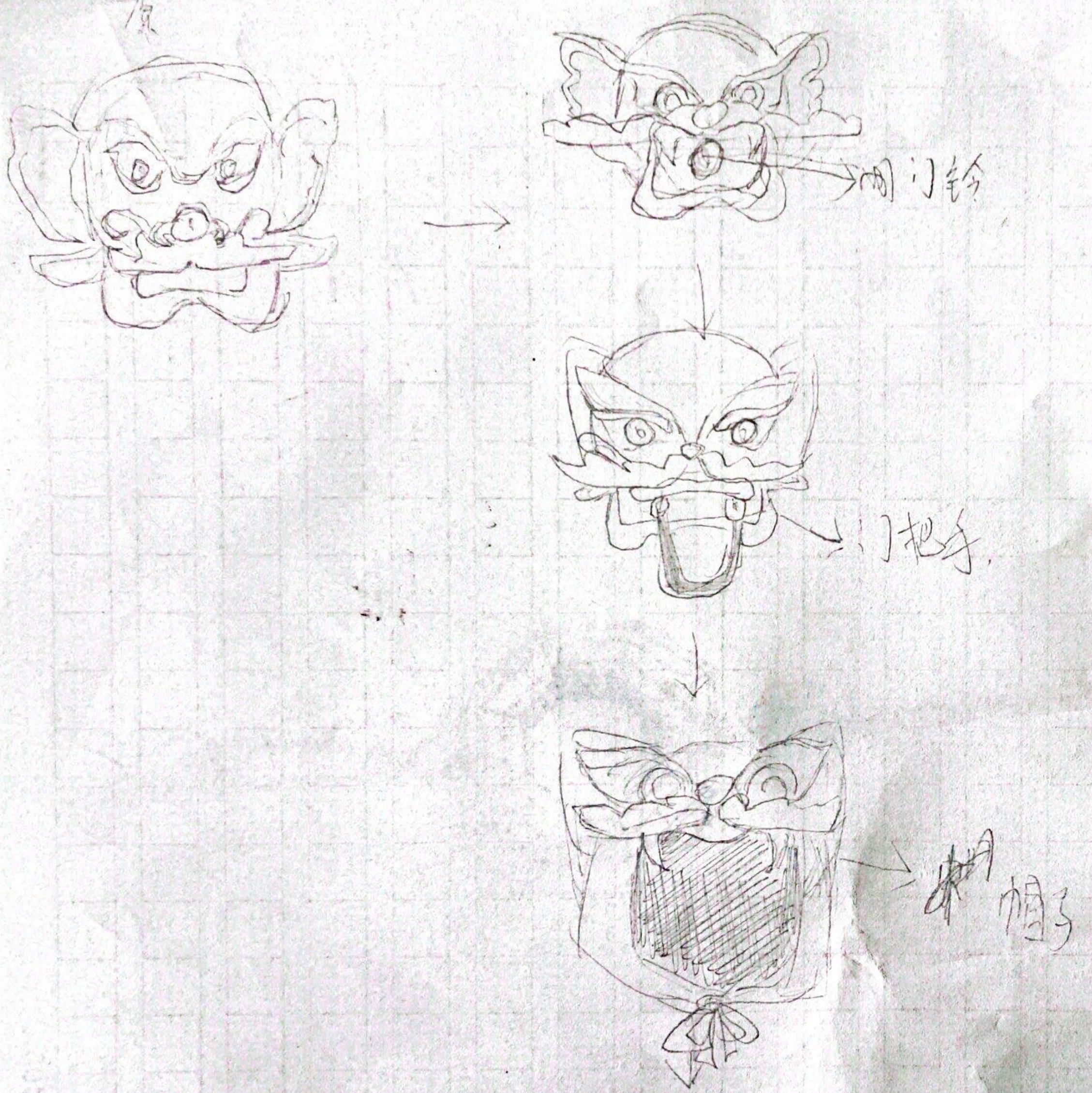

172002153

石[illegible]

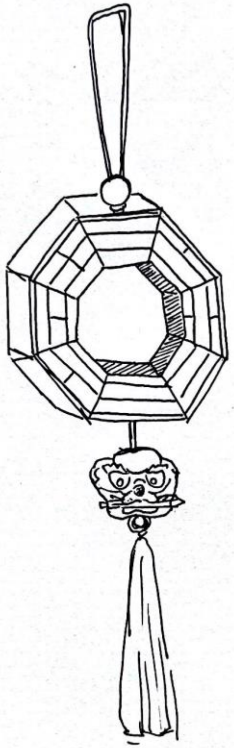

顾弈. 17202173.

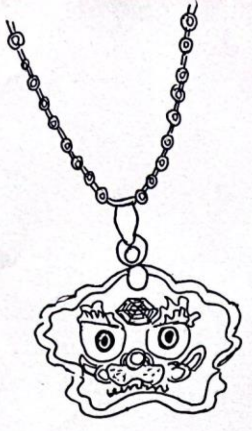

链玉坠.

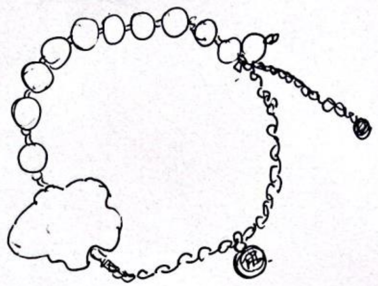

佛手链手绳

17002171

王一冰

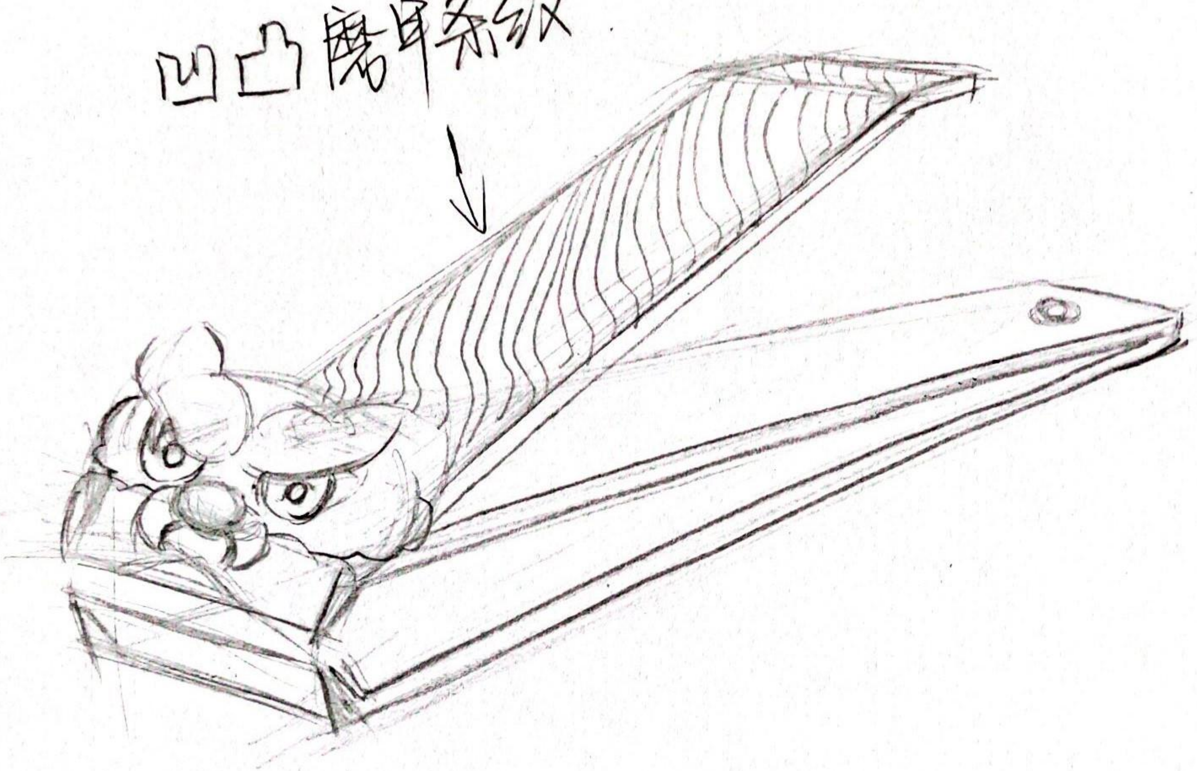

指甲刀.

林雨婷.
172002170.

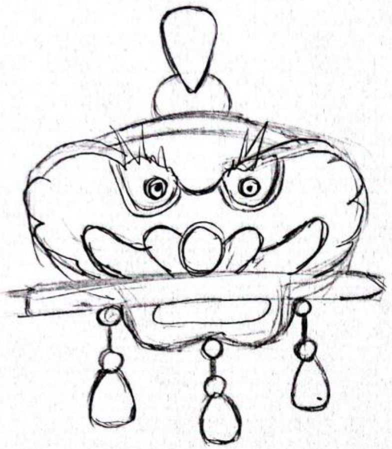

平安锁

周子璇
172002162

Utilize the design scheme generated by AIGC

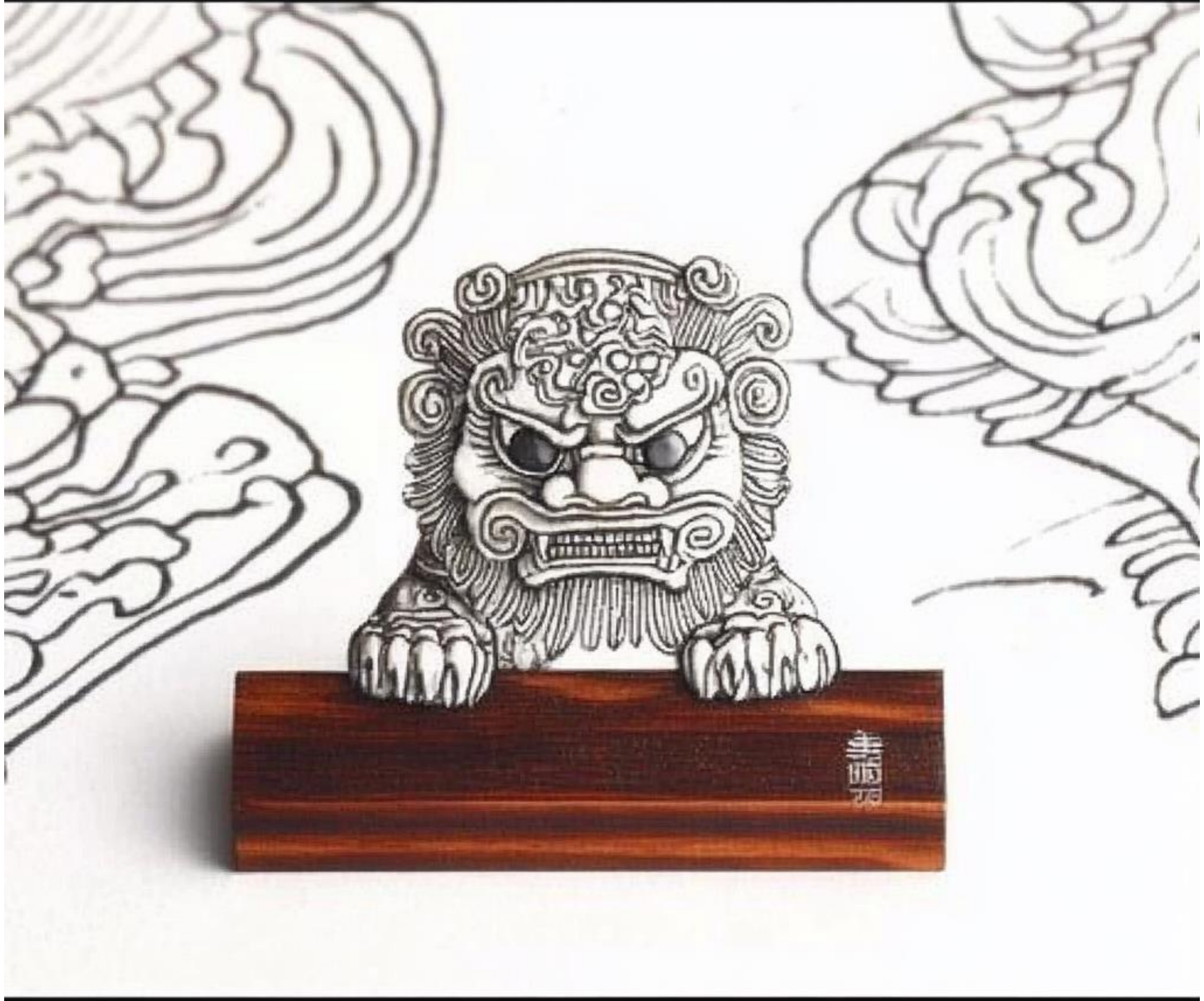

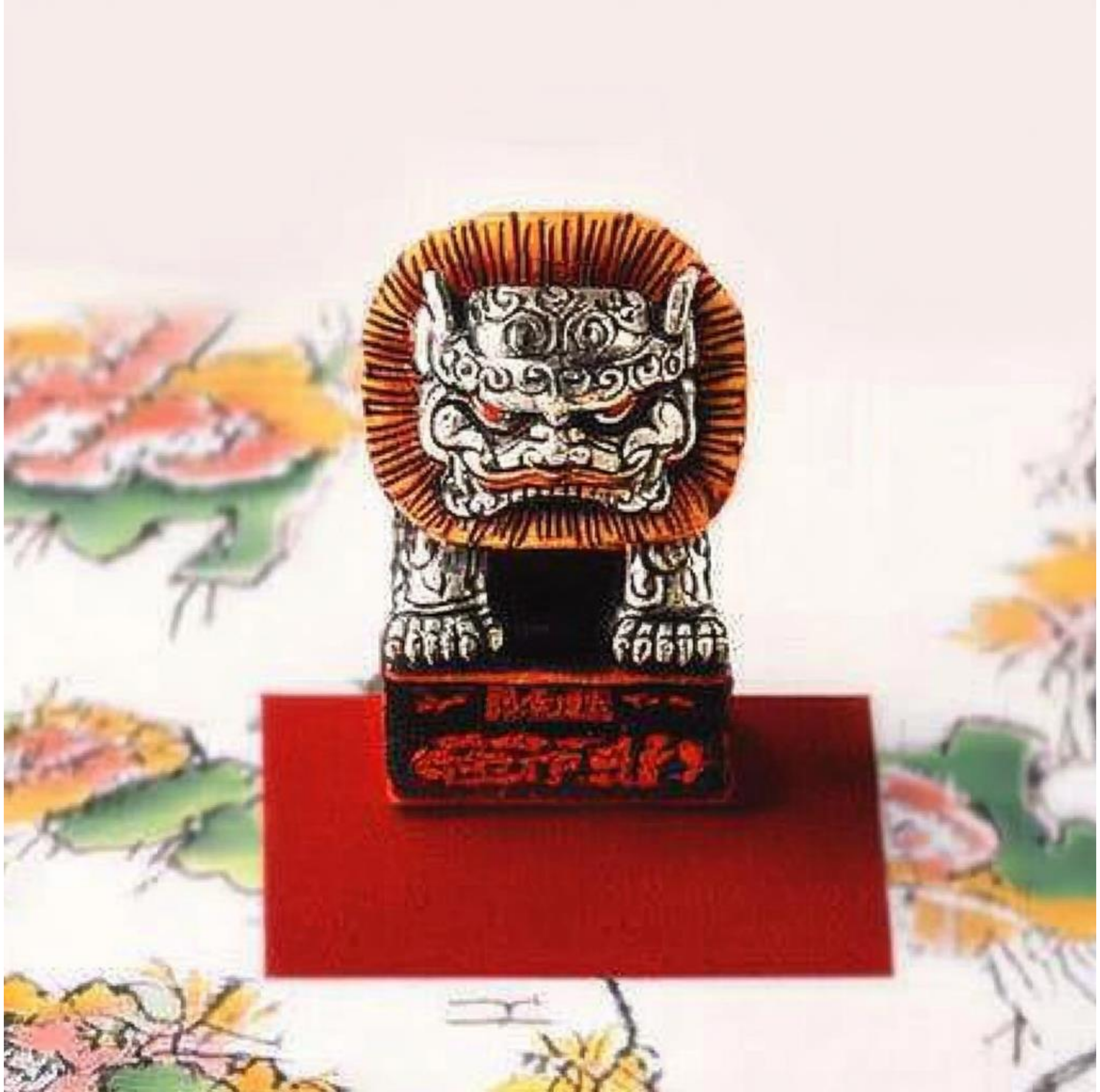

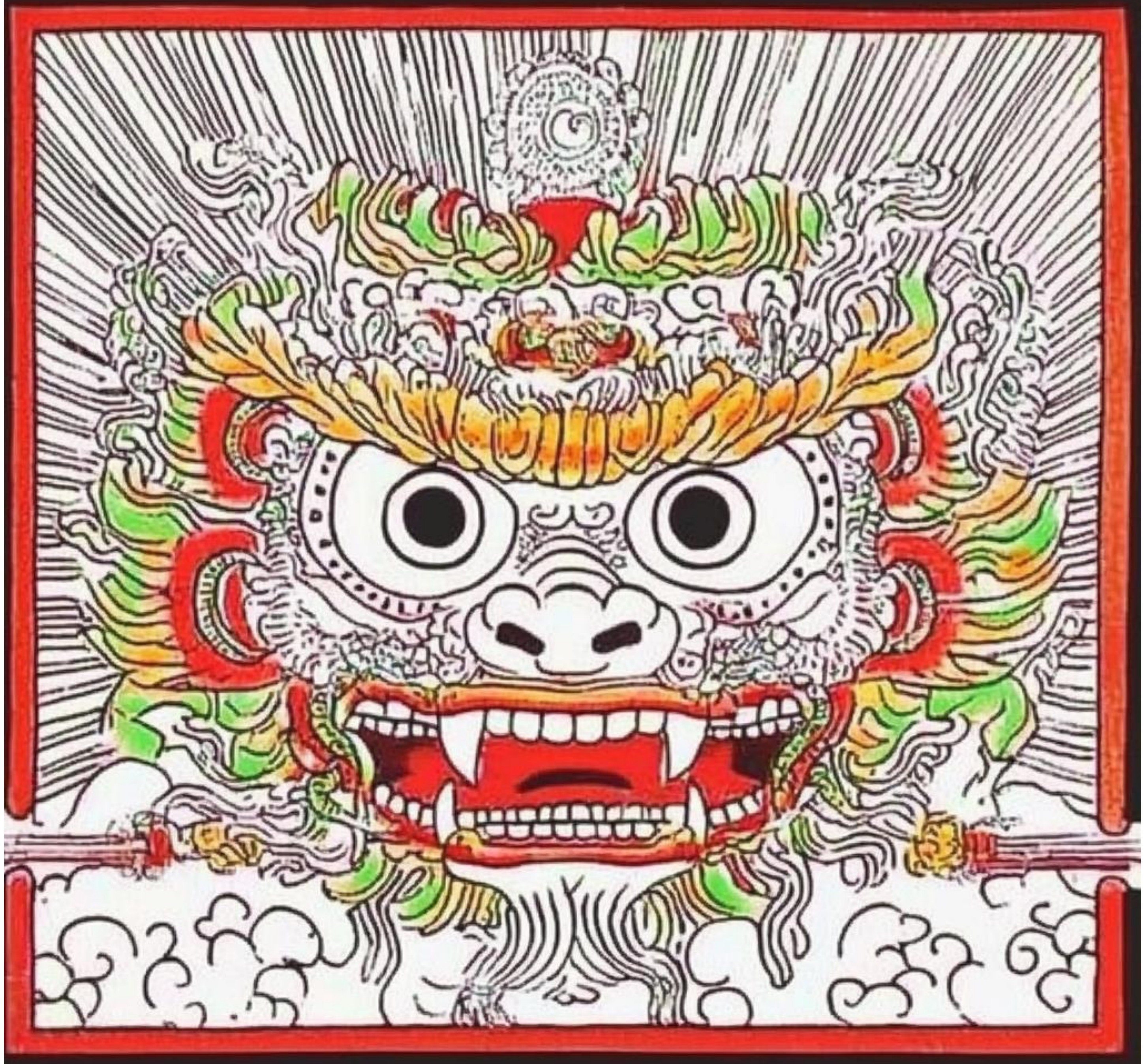

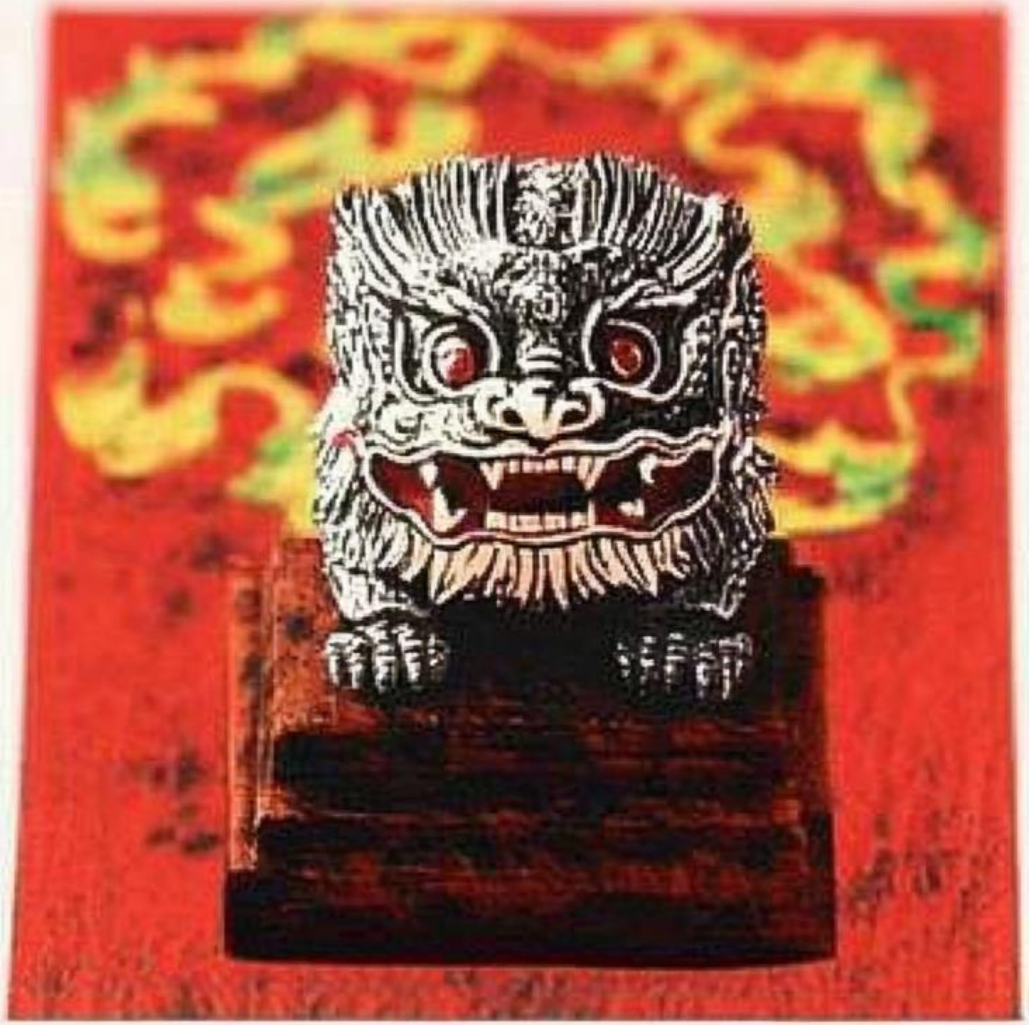

[illegible]

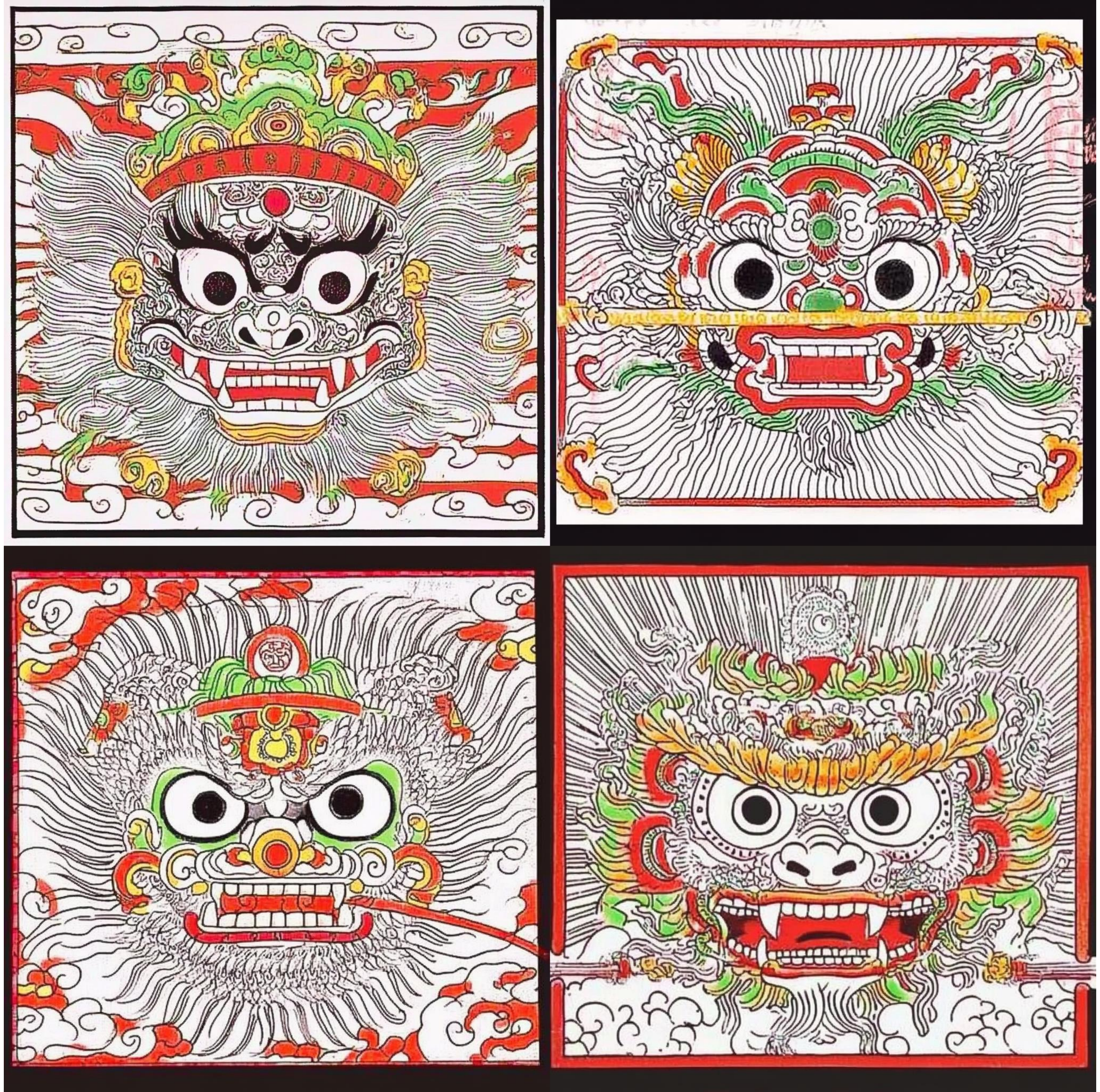

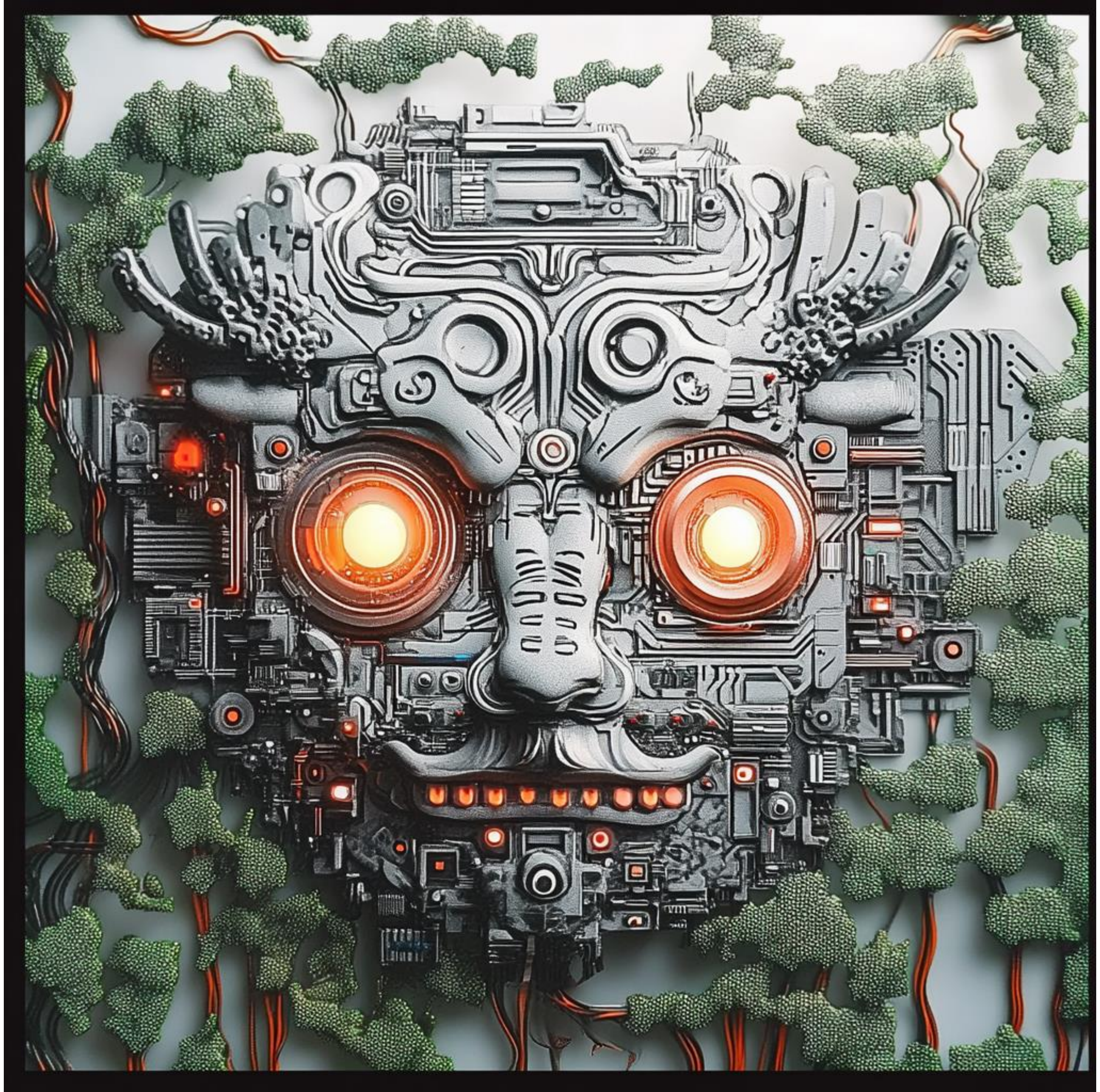

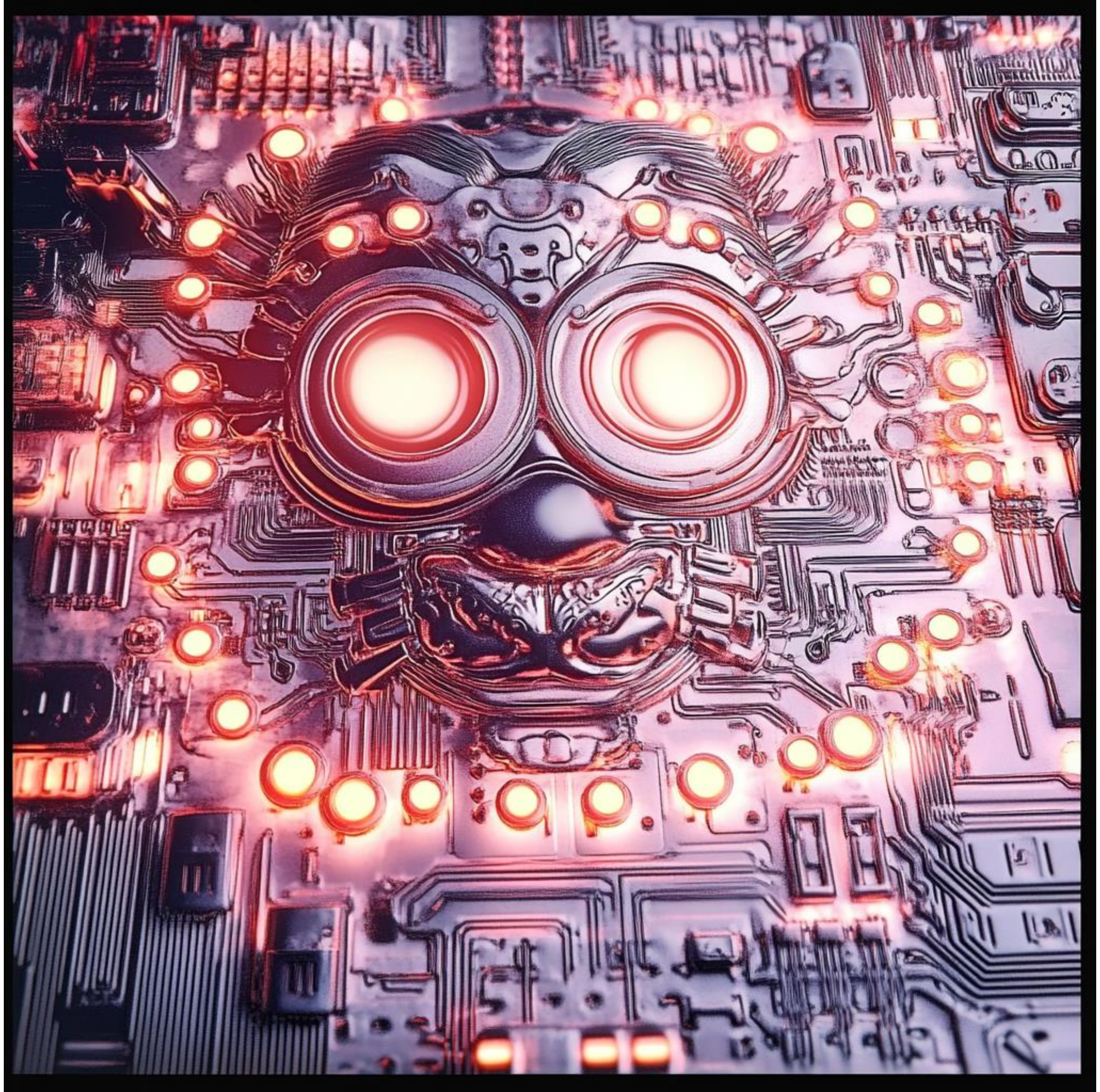

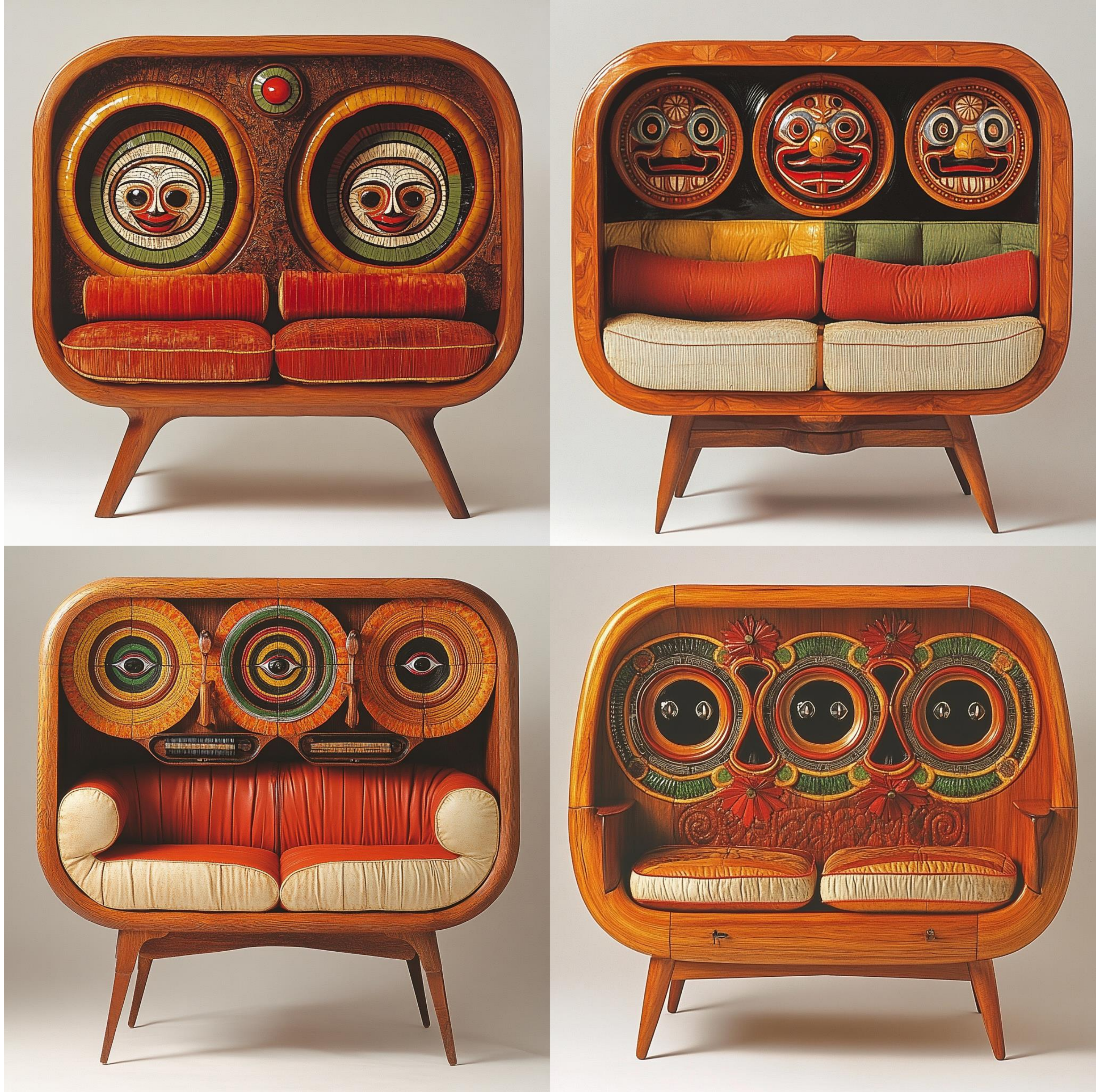

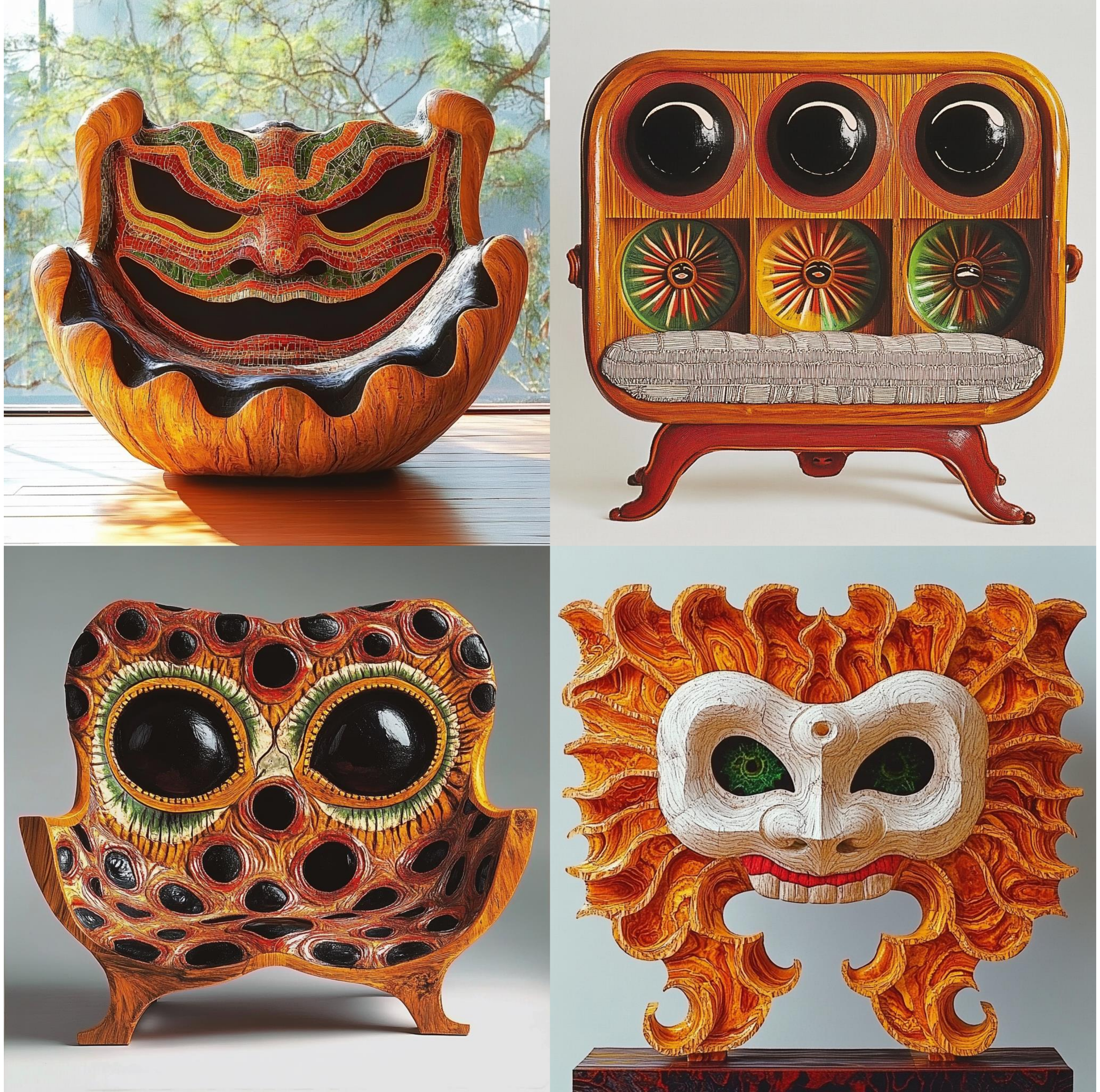

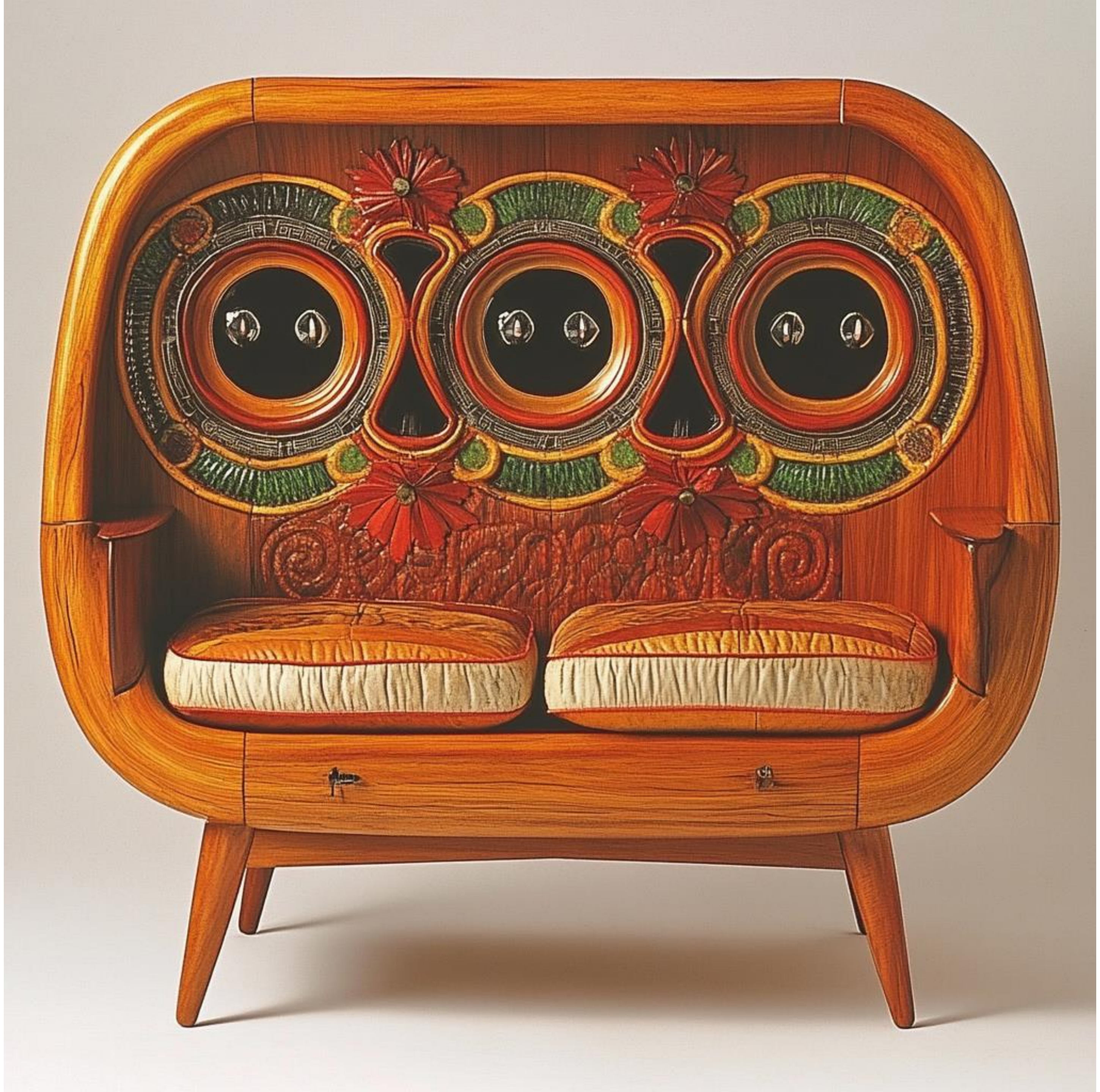

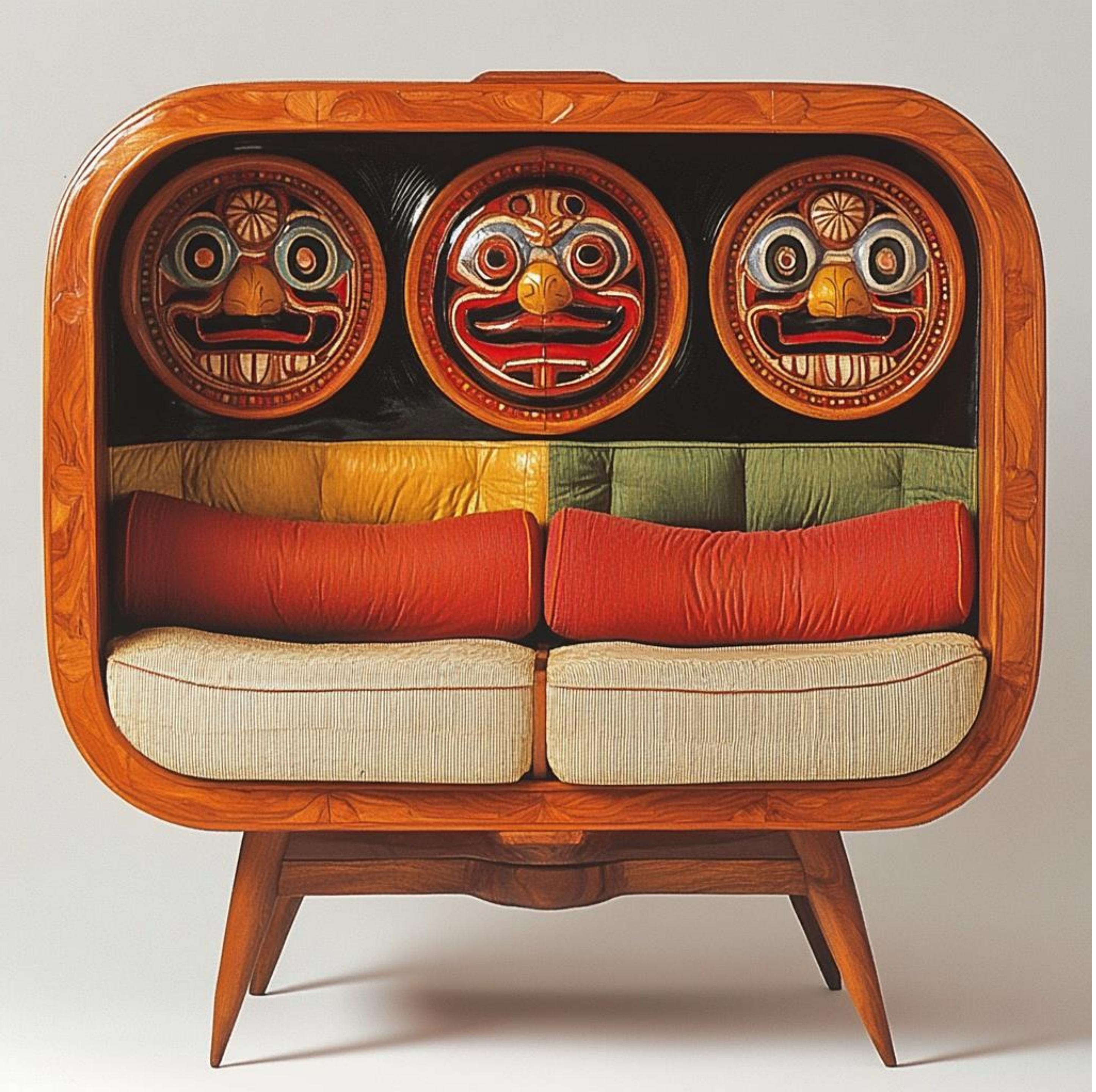

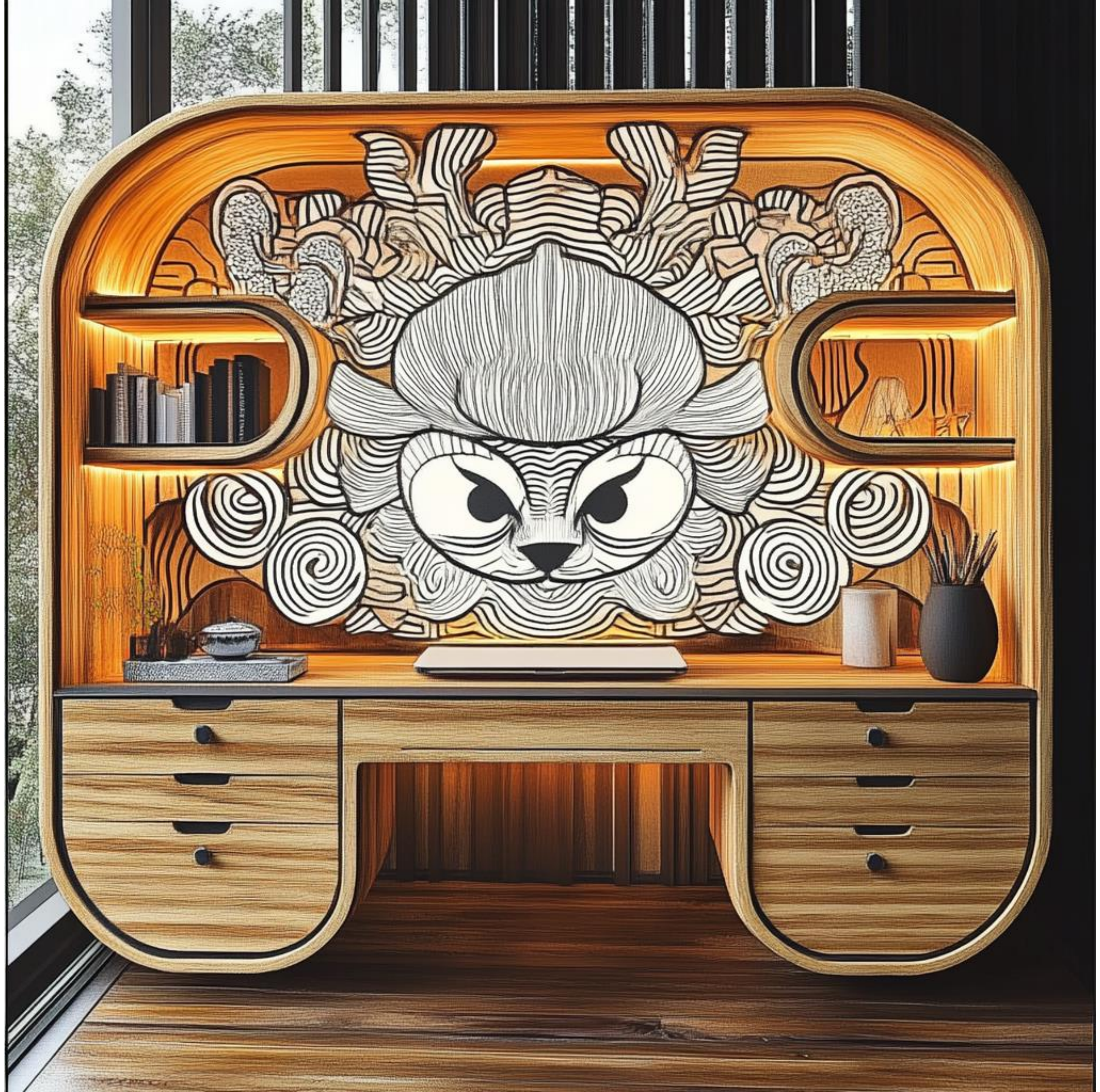

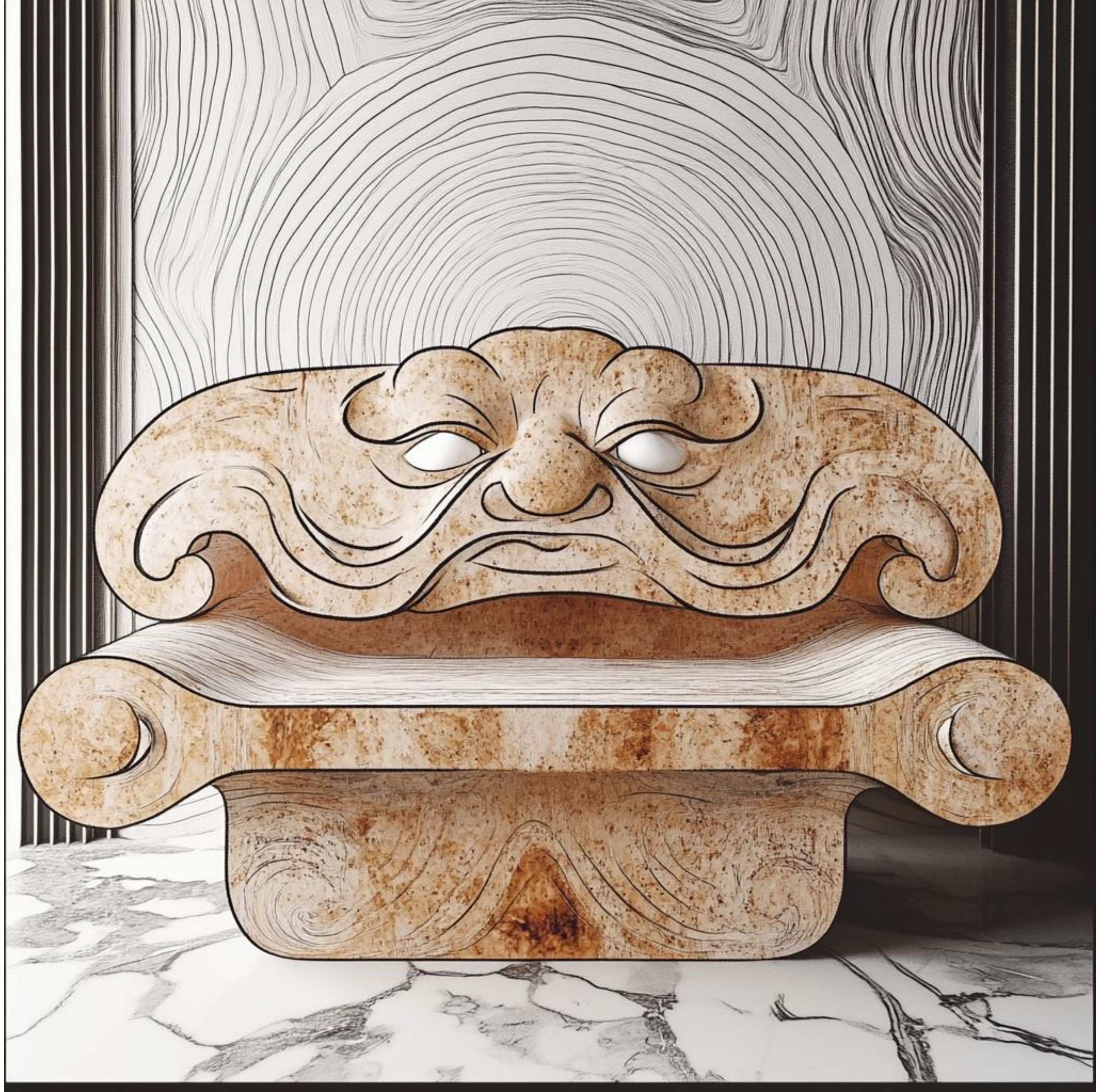

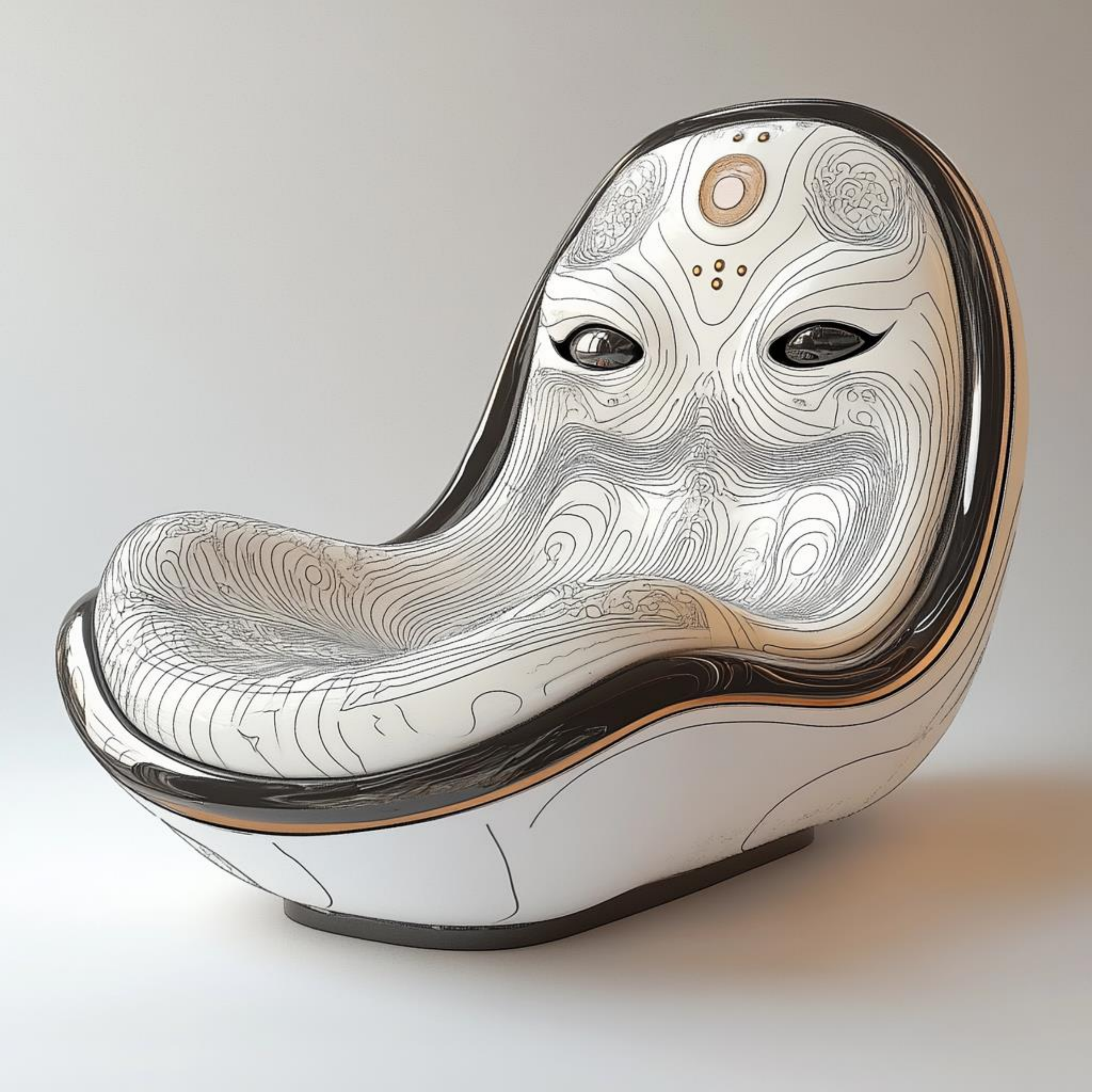

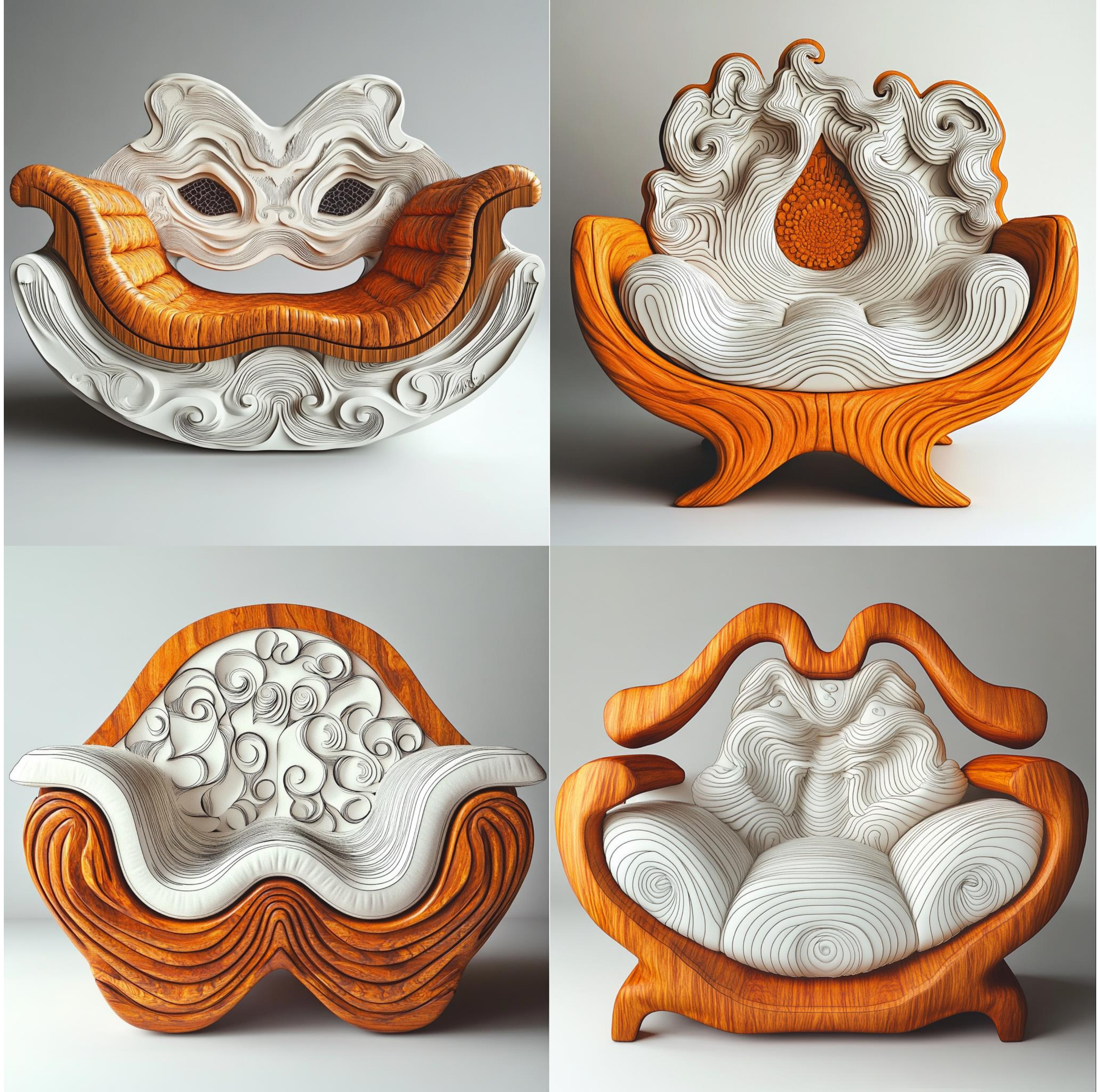

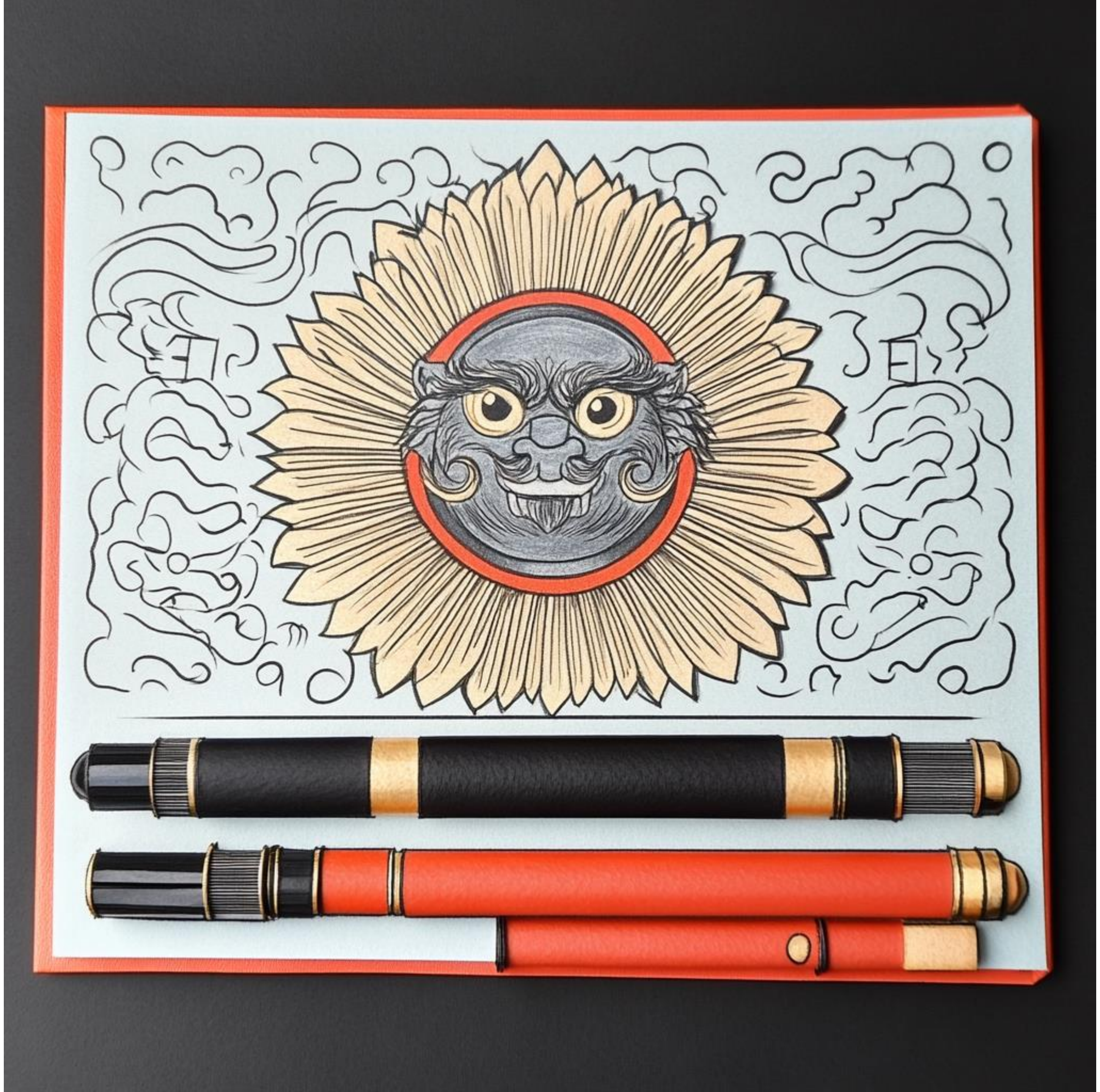

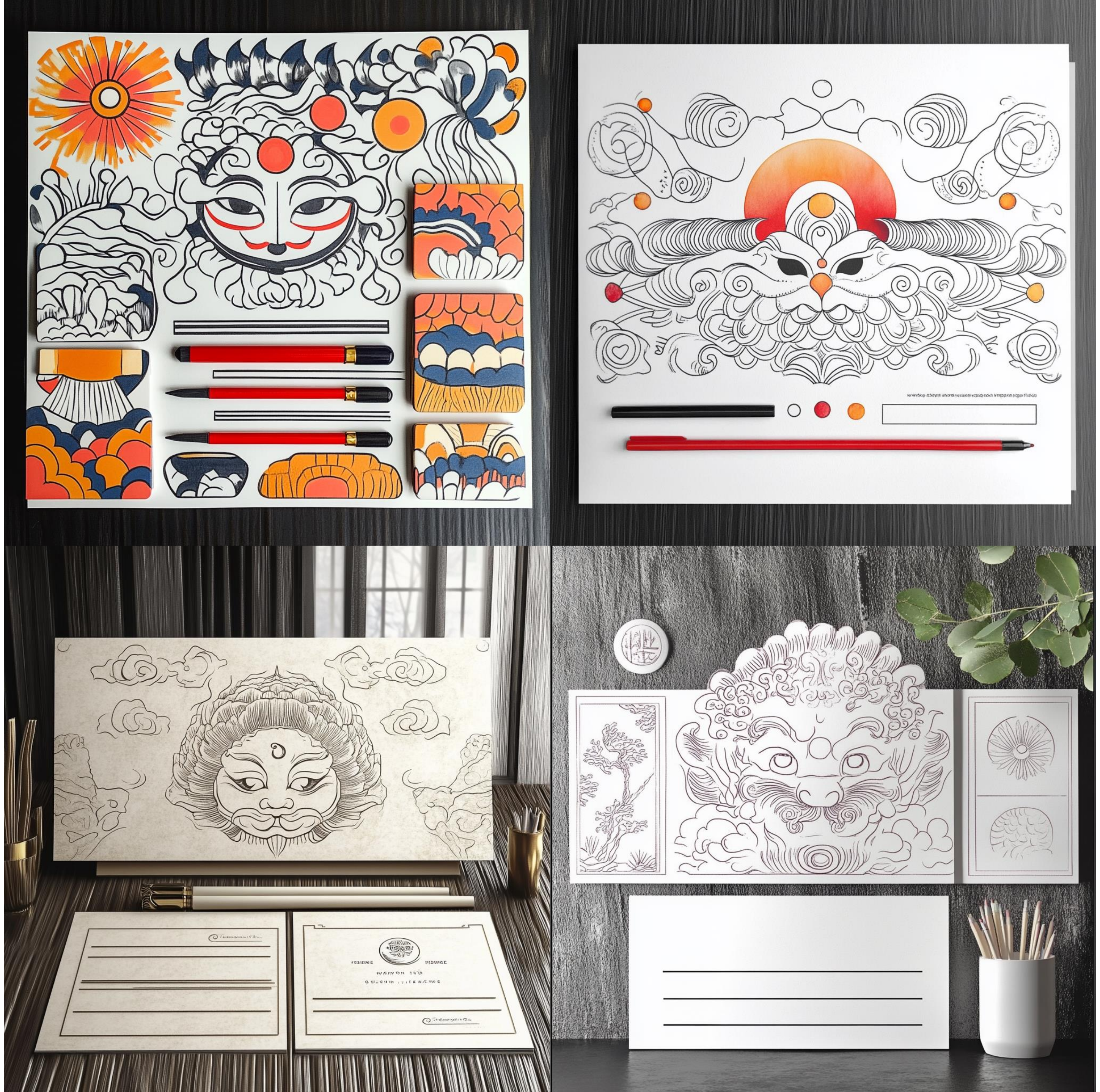

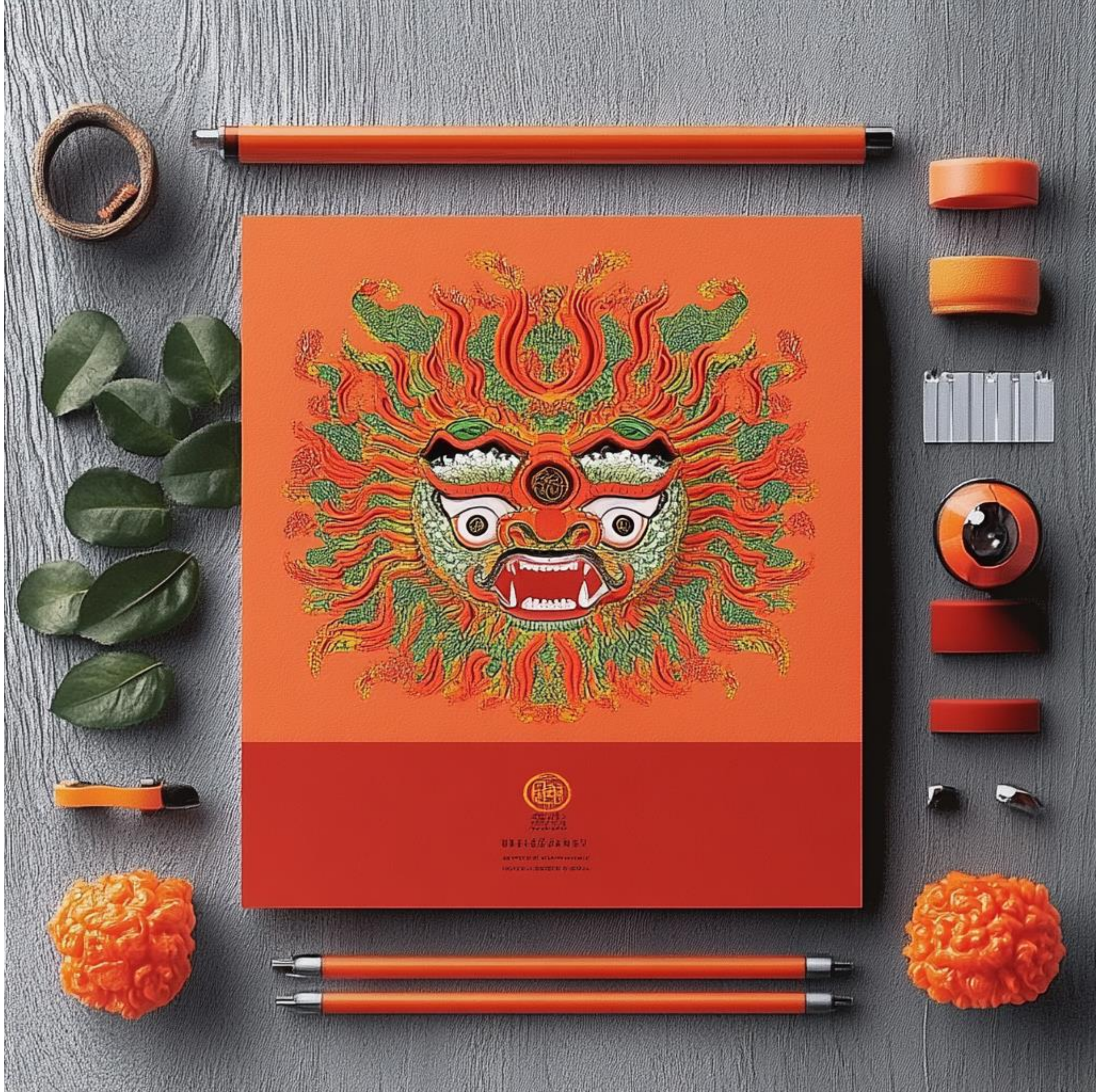

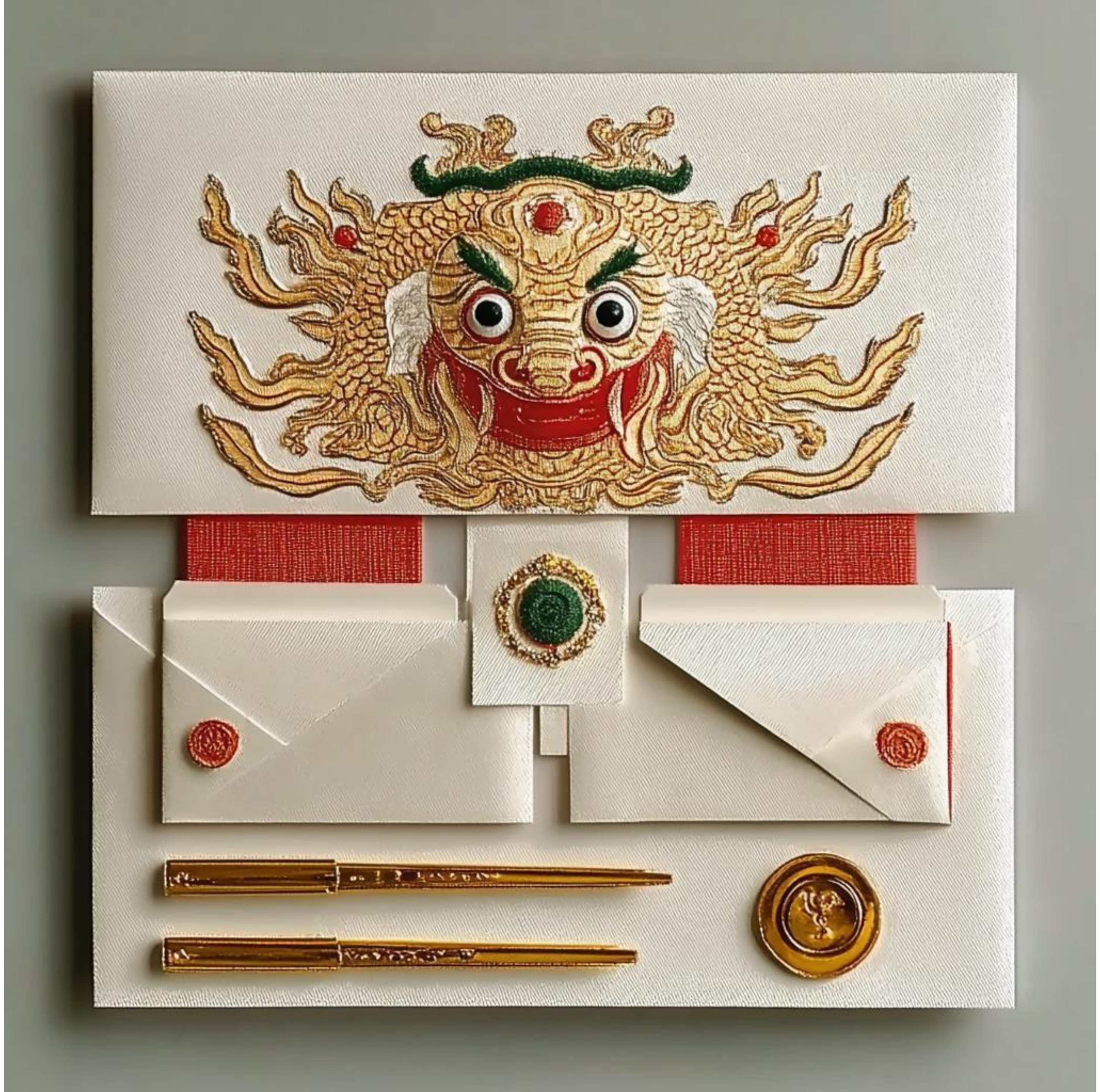

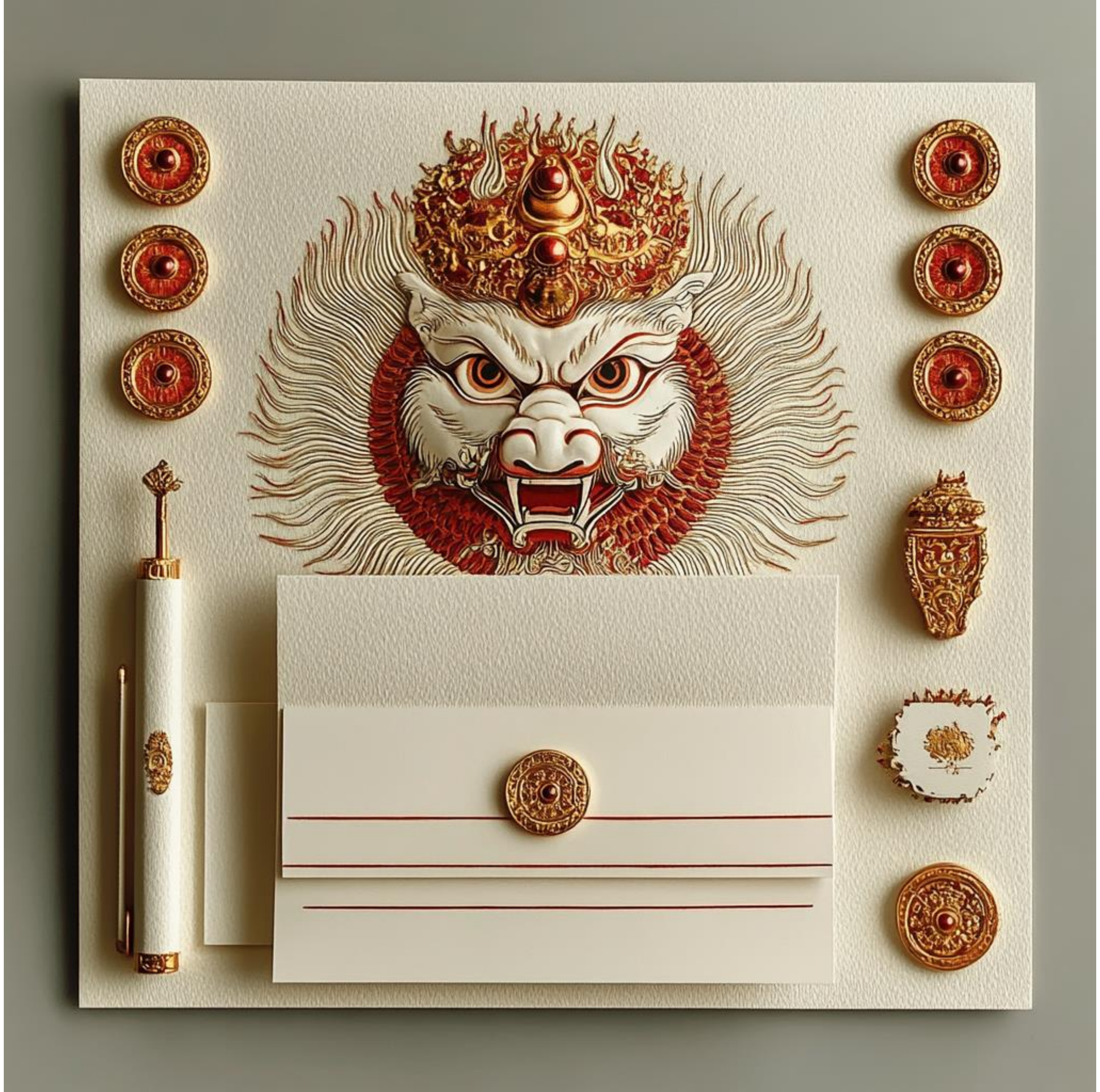

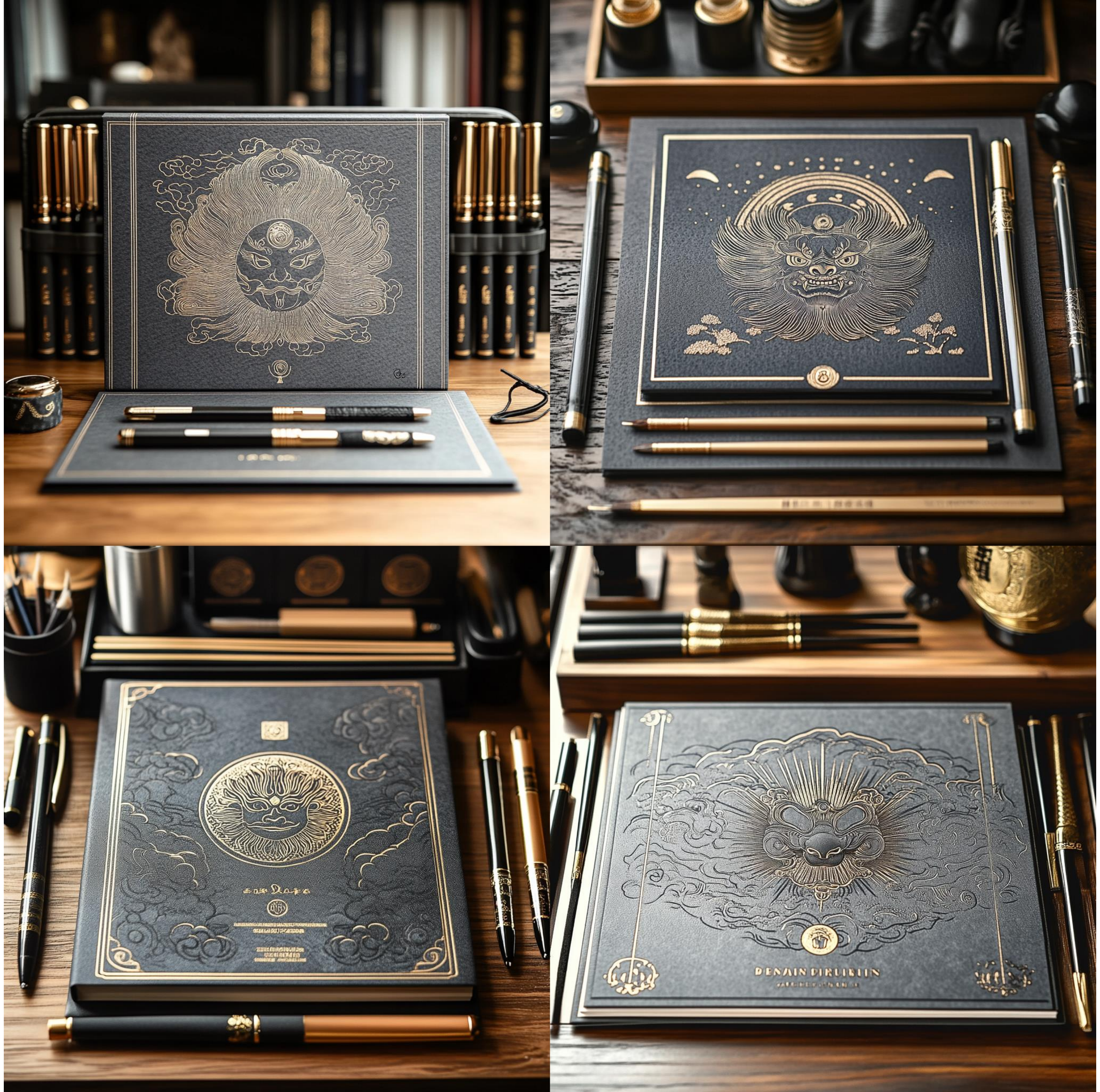

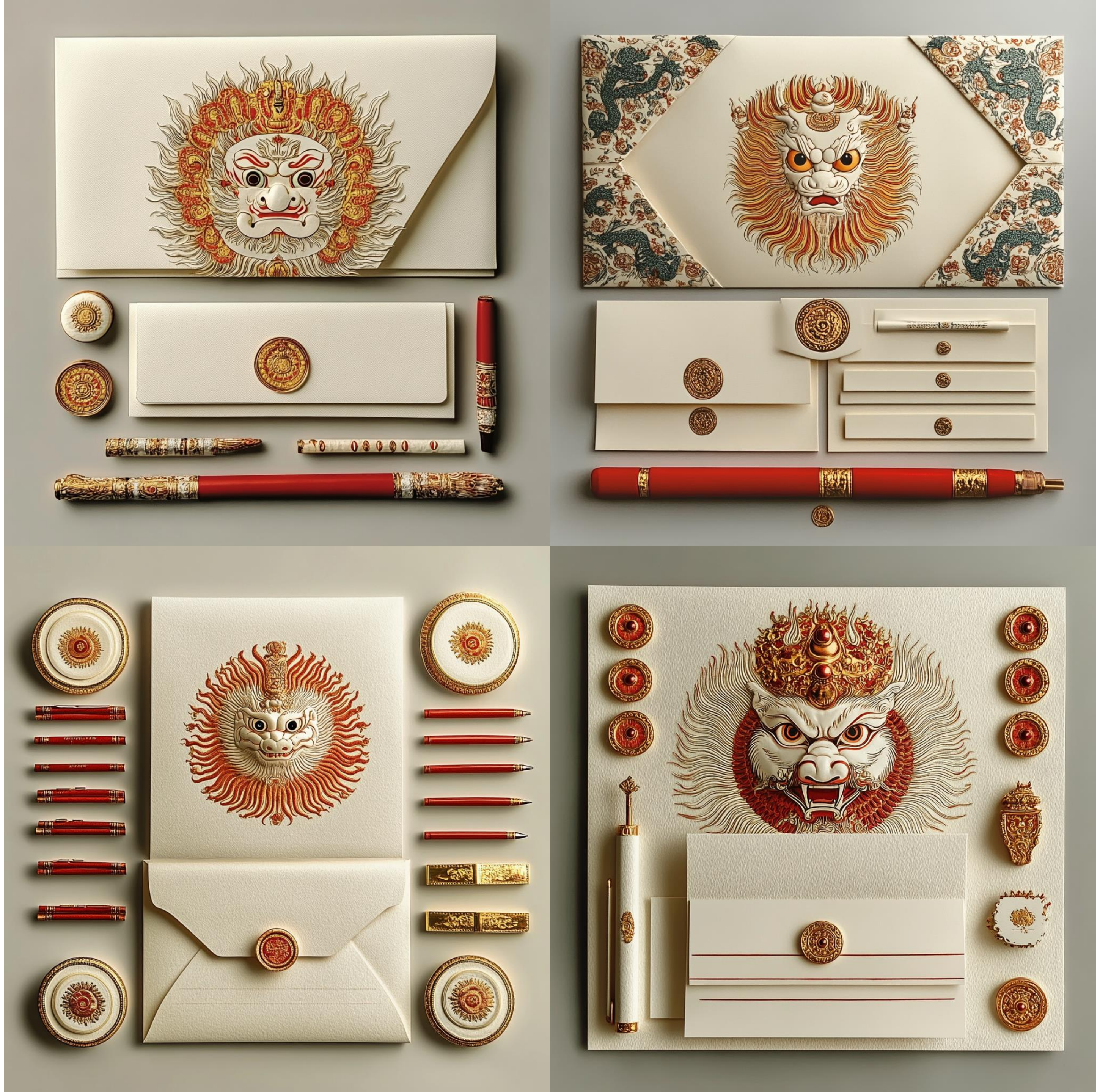

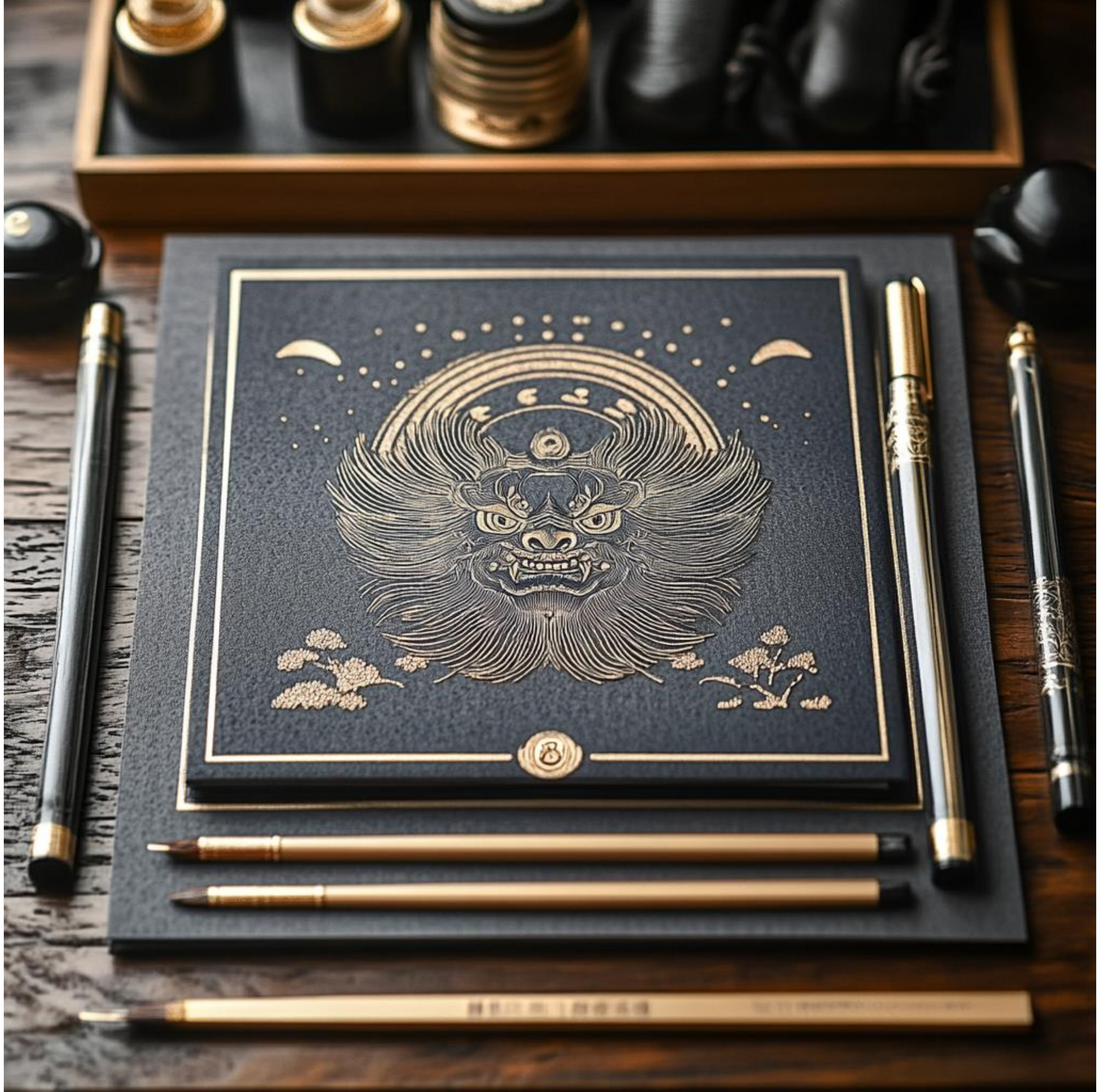

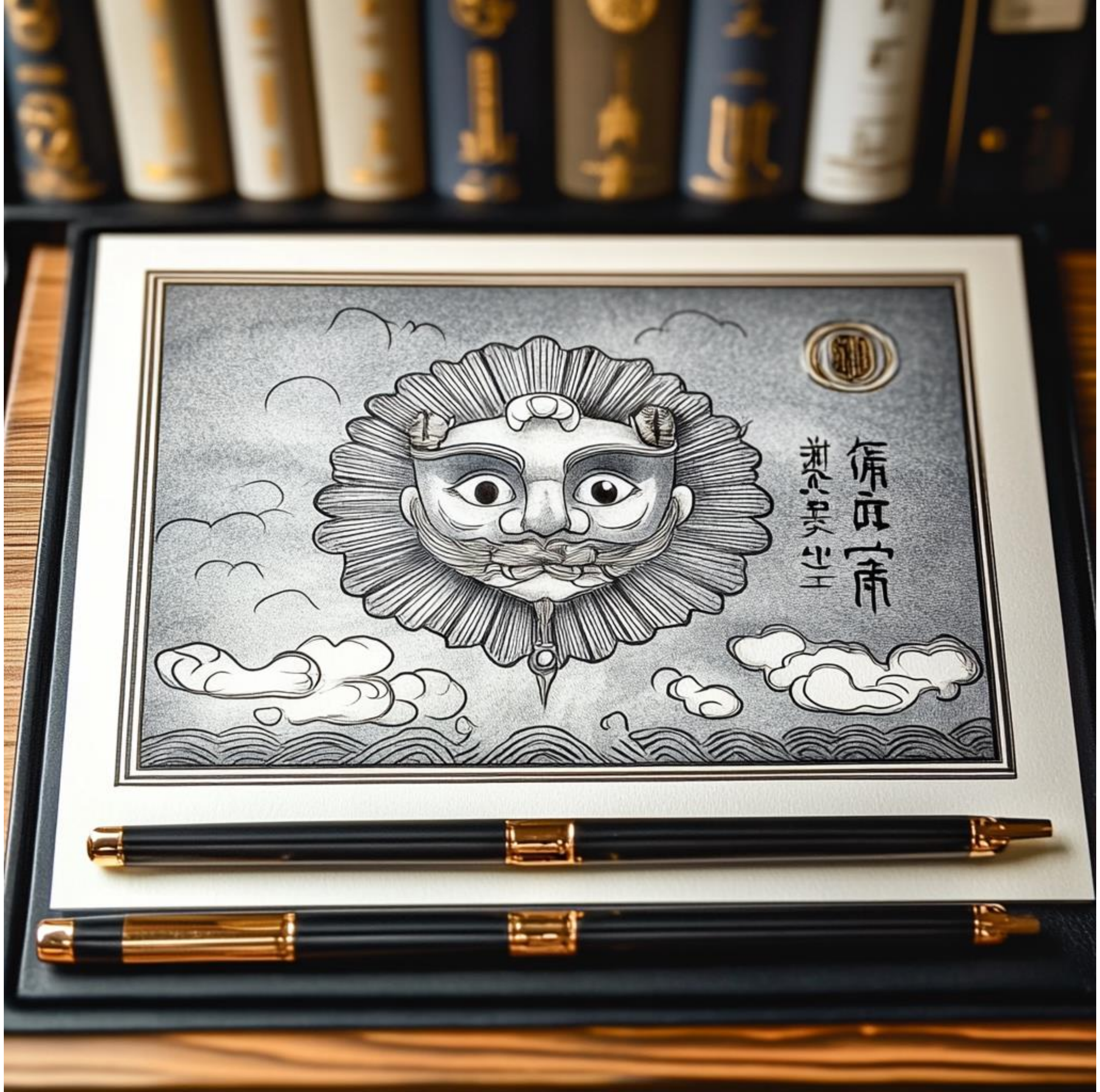

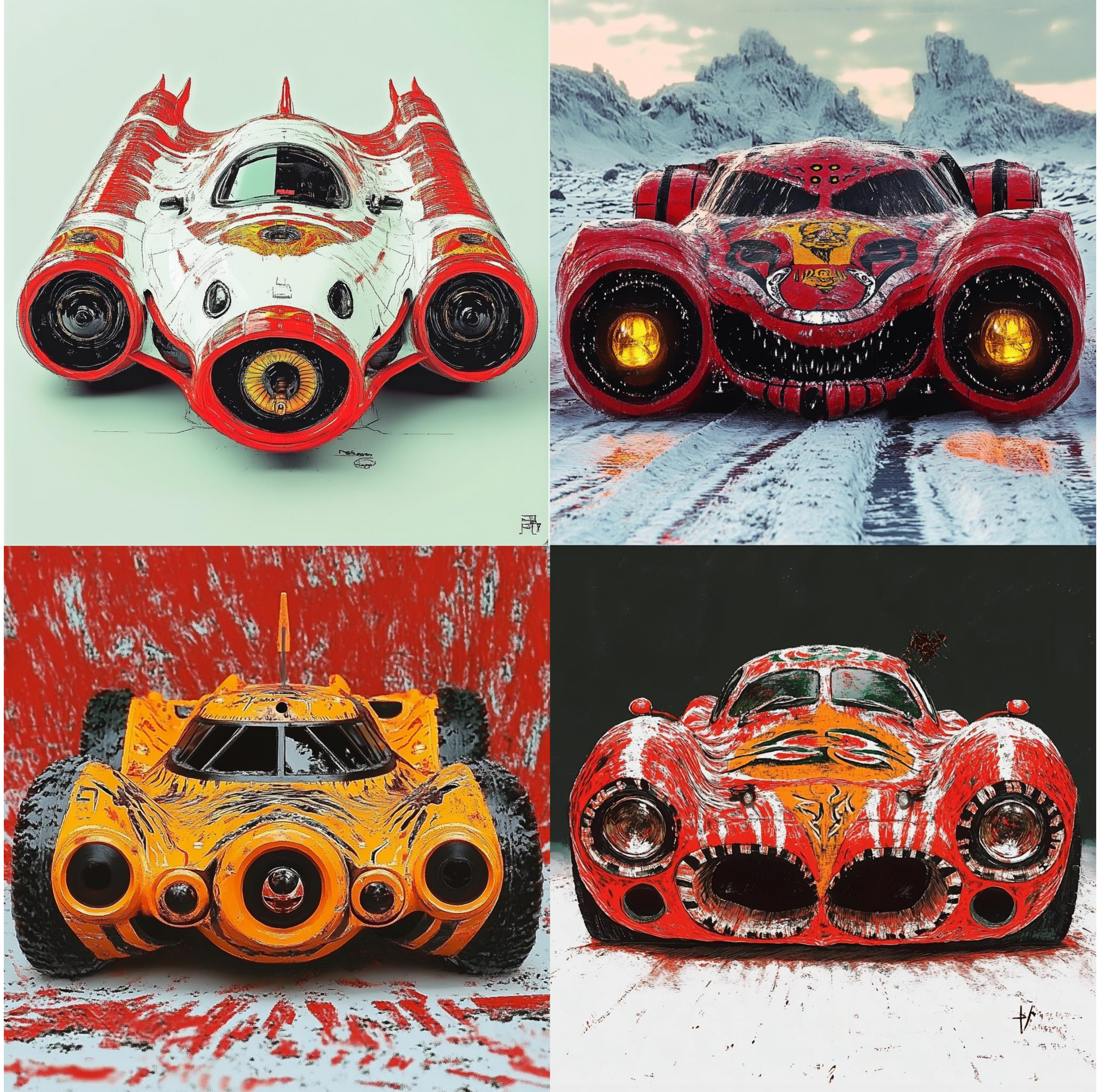

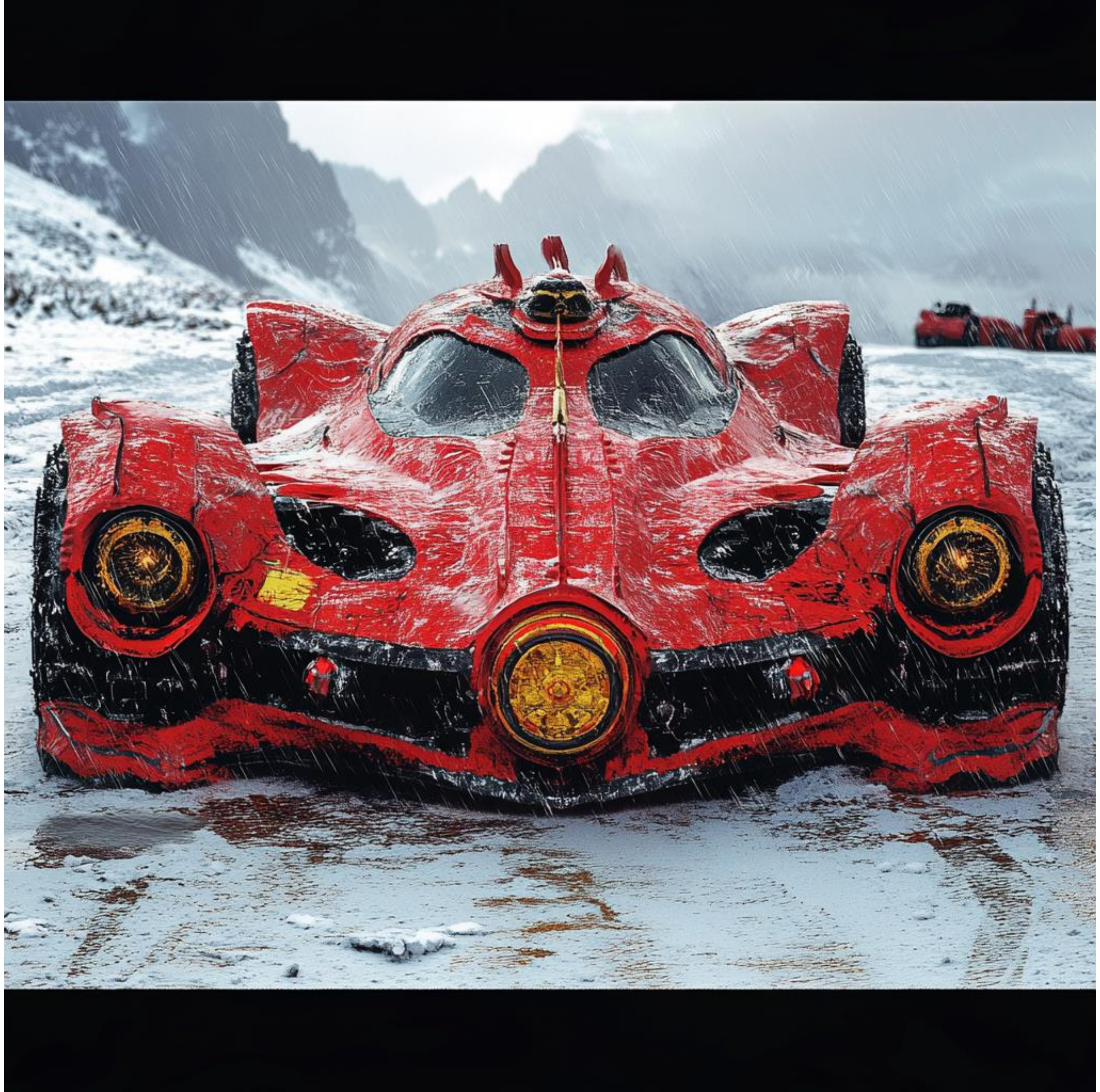

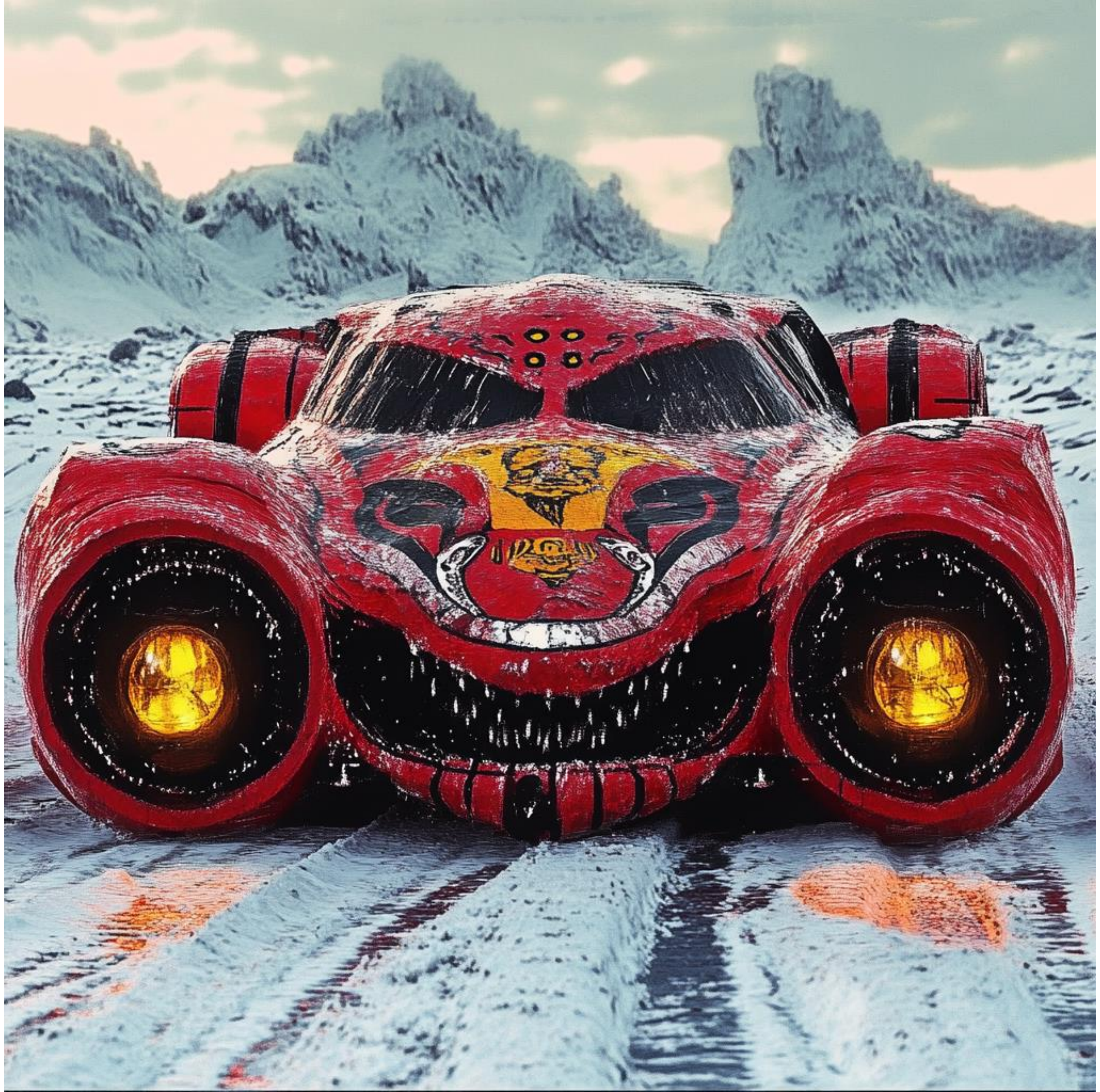

THANK YOU
