# Supplementary material for: Revitalizing Intangible cultural heritage via derivative design: A focus on chinese woodblock printing
Source: PLoS One. 2025 Apr 17;20(4):e0318807. doi: 10.1371/journal.pone.0318807 (PMC12005565; doi:10.1371/journal.pone.0318807)
Supplement: Attachment — Submitted filename: Response to Reviewers.docx [file pone.0318807.s004.docx]

Dear Editors and Reviewers:

Thank you for your letter and for the reviewers’comments concerning our manuentitled “Revitalizing Intangible Cultural Heritage via Derivative Design A Focus on Chinese Woodblock Printing”([PONE-D-24-04455] - [EMID:b43e276b40aaedd0]). Those comments are all valuable and very helpful for revising and improving our paper, as well as the important guiding significance to our researches. We have studied comments carefully and have made correction which we hope meet with approval. Revised portion are marked in red in the paper.

The main corrections in the paper and the responds to the reviewer’s comments are as flowing:

Responds to the Editor’s comments:

**1.** Response to comment: Please provide additional details regarding participant consent.

**Response:** This study is conducted as a routine part of regular teaching activities, with the experimental procedures forming an integral component of the curriculum. Oral consent has been obtained from all participants. The instructional content and processes have been ethically reviewed and approved by the ethics committee, obviating the need for additional documentation.

**2.** Response to comment: Data Availability Statement

**Response:** All data generated or analyzed in the course of this study are included in this published article and its supplementary file.

**3.** Response to comment: Additional corresponding author's ORCID iD

**Response:** Thank you very much for your reminder. I have added all the information.

**4.** Response to comment: Copyright confirmation of the image of Figure(s) 6, 7 and table 3.

**Response:** All images presented in this study are original creations by the author, including the contents of Figures 6 and 7, as well as Table 3 (revised as Table 4). As a design professional and educator, Figure 6 illustrates a synthesis of derivative design processes and original case studies. Figure 7 represents design practices conducted by the author based on the design methods proposed in this paper. The images in Table 3 (revised as Table 4) are also original creations by the author. There are no copyright issues involved. Thank you for your attention.

Thank you, editor, for your meticulous guidance and assistance. Your suggestions have been invaluable to me! In addition, this study added 21 new references. Notably, the content of the introduction, discussion, and experimental design sections has been expanded, adding a total of 2,300 words to the entire text. However, I am sorry that I cannot find the submission template of PLOS. I need your professional guidance and help in format adjustment.

Responds to the reviewer’s comments:

**Reviewer #1:**

**1.** Response to comment: The introduction lacks a clear statement of the main objectives of the study.

**Response:** Thank you to the reviewer for diligently revising this paper and providing detailed guidance on the author's revision process.

The primary objective of this research is to explore the feasibility and innovativeness of generative design in the protection and inheritance of Intangible Cultural Heritage. By employing literature review and experimental methods, this study investigates the advantages of generative design in terms of the quantity and quality of design proposals. From the perspective of design thinking, three derivative product design models are proposed, further enriching the methods of Intangible Cultural Heritage protection and pathways for its inheritance. **（Line151-156）**

**2.** Response to comment: The introduction lacks a clear articulation of the intended contribution to the existing literature.

**Response:**

**2-1.** Literature support for novelty

The practical application of Intangible Cultural Heritage in design innovation mostly remains at the stage of aesthetic form, lacking systematic innovation from the perspective of cultural elements and craftsmanship [3]. Current shortcomings in design innovation for Intangible Cultural Heritage protection are mainly reflected in the following aspects: (1) innovation only in form, without a profound understanding of the importance of cultural values[4]; (2) excessive pursuit of marketization and commercialization, leading to homogenization of existing products. While commercialization can expand the scope of dissemination, the single-minded pursuit of economic benefits weakens the original cultural and historical significance of Intangible Cultural Heritage [5]; (3) limitations of design innovation methods. The failure to adopt modern design thinking and tools leads to creative exhaustion[6]; (4) the limitations of dissemination media affect the range and acceptance of Intangible Cultural Heritage [7]。. In conclusion, to address the limitations of Intangible Cultural Heritage protection methods and dissemination channels, researchers should explore innovative thinking and methods from the root and apply them to specific cases for refinement and iteration. In the field of design, updating and applying design methods is the source of innovation. **（Line42-56）**

**2-2.** Innovation of this study

The innovations of this research are as follows: (1) Construction of a systematic design method. A new model for derivative product design is proposed, utilizing a method capable of generating diverse product design proposals in a short time; (2) Exhibition of diversified inheritance pathways. Various design carriers and methods are explored, including product design, digital design, and cultural innovation, enriching the design techniques and forms of Zhangzhou woodblock New Year picture derivatives; (3) Balancing innovative design with traditional inheritance. Through generative design, the works retain the characteristics of intangible cultural heritage while meeting modern aesthetic and practical needs. In summary, this research, from an industrial design perspective, explores the extraction and analysis of traditional elements and the integration of modern design methods to achieve a balance between traditional culture and contemporary design. **（Line157-167）**

**3.** Response to comment: More detailed description of the demographic characteristics of the control and experimental groups.

**Response:** To ensure the stability of the research, the control group and experimental group remained consistent across three stages of experiments, allowing for a scientific comparison of the facilitative effects of derivative design methods on the redesign of intangible cultural heritage. All participants have completed courses in "Traditional Cultural Redesign" and "Cultural Creative Design," enabling them to proficiently utilize design software and manual sketching for design expression. The basic information of the subjects is presented in Table 1. **（Line471-476）**

**Table 1.** Basic information of interviewee (N = 46)

| Profile | Items | Number | Percentage(%) |
| --- | --- | --- | --- |
| Gender | Male | 16 | 35% |
|  | Female | 30 | 65% |
| Age | 20 | 6 | 13% |
|  | 21 | 32 | 70% |
|  | 22 | 8 | 17% |
| Education | Junior | 46 | 100% |
| Major | Product design | 46 | 100% |
| Design Experience | Yes | 46 | 100% |

**4.** Response to comment: Experimental setup, particularly regarding participants' awareness of their involvement and measures taken to mitigate the Hawthorne effect, need further explanation to enhance the study's validity.

**Response:**

Phase One: Cultural Creative Product Design Inspired by Different Intangible Cultural Heritage

Due to the diverse types of intangible cultural heritage, there are significant variations in their forms and design approaches. This study selects "Quanzhou Nanyin" representing auditory stimulation, "Zhangzhou Woodblock New Year Pictures" representing visual stimulation, and "Ming-style furniture craftsmanship" representing tactile stimulation as sample stimuli from a sensory perspective. Design students participating in the practice are tasked with innovatively designing derivative products based on these stimuli. Ultimately, it was found that the highest number of design proposals originated from the inspiration of "Zhangzhou Woodblock New Year Pictures" (visual stimuli). This suggests that visually inspired designs have an advantage in terms of the quantity of derivative products generated.

Phase Two: Training on Generative Design for the Experimental Group

This phase divides participants into an experimental group and a control group, each comprising 23 individuals. The experimental group undergoes systematic training in generative design methods, while the control group receives no training. Results indicate that designers trained in generative design can produce a greater quantity and higher quality of design pro-posals.

Phase Three: Cultural Creative Design Proposition Inspired by "Zhangzhou Woodblock New Year Pictures"

This phase aims to further explore the application value of generative design in design practice, focusing on deriving methods for designing products inspired by "Zhangzhou Wood-block New Year Pictures." Research results demonstrate that generative design can systematically propose derivatives for intangible cultural heritage, enriching modes of preservation and trans-mission. Detailed experimental data and results will be presented in the following sections. **（Line482-504）**

**5.** Response to comment: Furthermore, the last two columns in Table 1 have the same heading. I suspect the rightmost heading is incorrect. The author should review it carefully.

**Response:** Thank you for pointing out the error, which was due to inappropriate translation. After consulting with experts, the appropriate title for the rightmost column in Table 1 is "Protection model innovation." I appreciate the reviewer's feedback in identifying this oversight. **（Line 446**- Fig 5.**）**

**6.** Response to comment: It is necessary to provide a detailed introduction to the VOSViewer platform to ensure accessibility for all readers.

**Response:** VOSviewer can visualize the research history and hotspots in this field, providing direction for research trends and focal points. VOSviewer, developed by the Centre for Science and Tech-nology Studies at Leiden University, is an open-source software tool that uses text mining of titles, authors, and keywords for term frequency, co-occurrence, and co-citation analysis. It aids re-searchers in visualizing bibliometric networks, commonly used to construct and visualize bibli-ometric maps within scientific domains, thus presenting research history and trends [20]. Its main functionalities include: (1) constructing co-occurrence, citation, and co-citation maps; (2) visualizing bibliometric networks; (3) text mining of literature to uncover research hotspots. Overall, VOSviewer is widely used in research evaluation, analysis of academic frontiers, and the construction of knowledge maps, providing robust support for understanding research back-grounds and trends [21]. This study employs VOSviewer for literature mining and utilizes visual maps to illustrate historical documents and research trends. **（Line266-277）**

**7.** Response to comment: Furthermore, specific terms and descriptions in the paper, such as "Yan value" economy need further clarification.

**Response:** The English translation for " Yan value economy " should indeed be "Beauty Economy." I apologize for any confusion caused by my limited proficiency in English. Thank you for clarifying. Please provide the specific literature references and application descriptions for further assistance.

Transforming traditional intangible cultural heritage into modern derivative products through commercialization is an important means of preservation. Among these, the aesthetic appeal of the derivatives is a crucial factor. American economist Daniel Hamermesh, in his paper "Beauty and the Labor Market," pointed out that there is both an "Ugliness Penalty" and a "Beauty Premium" in society, indicating that physical attractiveness is positively correlated with overall labor income[10]. This preference for beauty extends to individuals, cultural artifacts, and commercial products [11].

The term "Beauty Economy" refers to the phenomenon where, in the context of product homogenization, users are more inclined to purchase products that offer aesthetic appeal and a superior user experience[12] This paper introduces this concept into the protection of Intangible Cultural Heritage, aiming to increase young people's interest in traditional culture through product innovation and to enhance the commercialization of Intangible Cultural Heritage through the power of design[13]. **（Line102-114）**

**8.** Response to comment: Was the process of creating woodblock New Year pictures in Figure 5 an original contribution or an adaptation of existing research? If the latter, appropriate citations are needed from existing literature.

**Response:** The process of creating woodblock New Year pictures in Figure 5 is original to the author. The author synthesized this craft process through field visits to museums in Zhangzhou, Fujian Province, interviews with intangible cultural heritage inheritors, literature review, and collection of videos and images. The production process of Zhangzhou woodblock New Year pictures is depicted in a concise and comprehensible process diagram. **（Line 446**- Fig 5.**）**

**9.** Response to comment:The discussion on the protection of Chinese intangible cultural heritage and its impact on economic activities and cultural innovation involves a critically significant field. However, further engagement with authoritative literature in this area is necessary.

**Response:** Firstly, I appreciate the careful guidance from the reviewer. The references you provided have deepened my understanding of China's policies and methods for safeguarding intangible cultural heritage. Through the literature review process, I thoroughly studied "China’s emerging legislative and policy framework for safeguarding intangible cultural heritage" and "Cultural impacts of state interventions: Traditional craftsmanship in China’s porcelain capital in the mid to late 20th century." Here is a detailed summary of China's supportive policies and the effectiveness of its efforts in protecting intangible cultural heritage:

2.1. Research status of Chinese intangible cultural heritage

The Chinese government has been protecting religious and related intangible cultural her-itage through various measures such as the intangible cultural heritage protection list, intangible heritage inheritors, and cultural innovation [17]. Notably, in 2006, the first batch of the intangible cultural heritage list was announced, and protection has been achieved through recognition mechanisms, talent cultivation, and demonstration bases[1]. These efforts have yielded both macro and micro-level results, as detailed below:

2.1.1. Macro Measures and Outcomes:

（1） Policy Formulation:Enhancing intangible cultural heritage protection through legal reg-ulations, thereby promoting cultural prosperity.

（2） Economic Benefits:Intangible cultural heritage contributes to the tourism economy, commodity economy, and city branding.

（3）Social Benefits:The protection and inheritance of intangible cultural heritage help reduce poverty and increase employment opportunities.

（4）Government Support for Regional Culture Promotion:Various religious groups have ex-panded their influence by showcasing, documenting, and promoting religious-related intangible cultural heritage [17].

These measures implicitly reflect innovative protection models and achievements, providing effective support for enriching the design of intangible cultural heritage derivatives. **（Line169-188）**

2.1.2. Micro Measures and Outcomes:

The Chinese government has numerous successful experiences in policy formulation and implementation:

（1）Productive Protection Model: Integrating cultural and economic development goals for mutual reinforcement.

（2） Multi-level Legislative Framework: Legal guarantees for intangible heritage protection through comprehensive regional policies.

（3） Collaboration between Government and Academia: Actively supporting scholars in identifying, documenting, and theorizing intangible cultural heritage information.

（4） Active Use of New Media Creation Platforms: Diversifying promotional channels and methods to enhance the influence and public participation in intangible cultural heritage protec-tion.

Overall, these innovative measures demonstrate China's comprehensive and diversified strategies for protecting intangible cultural heritage, encompassing legal protection, economic support, social participation, and new media dissemination[1].

Despite significant achievements in the protection policies and inheritance approaches for intangible cultural heritage, there are still issues such as limited protection methods, homoge-neous inheritance carriers, and outdated design innovation methods. This study will explore derivative design methods and applications for intangible cultural heritage from the perspective of design innovation, further enriching the methods and product carriers for heritage inheritance. **（Line189-208）**

Special thanks to you for your good comments.

**Reviewer #2:**

**1.** Response to comment: In the introduction, the author's research plan is challenging to comprehend.

**Response:** Thank you for the reviewer's meticulous guidance on the structure of this paper!

We rewrote the research objectives, innovations and processes as follows:

**1-1.** Research Background

Intangible Cultural Heritage (ICH) plays a crucial role in cultural diversity, social cohesion, and economic development. On one hand, protecting Intangible Cultural Heritage helps enhance cultural inheritance, national confidence, and tourism economy; on the other hand, it can also promote sustainable social and economic development [1]. However, the process of protecting and inheriting Intangible Cultural Heritage faces the following challenges: (1) conservative themes that fail to meet the needs of the new era; (2) the high difficulty of traditional craftsmanship hinders modernization; (3) severe industry homogenization affects cultural dissemination; (4) the scarcity of derivatives impedes commercial communication. Therefore, it is evident that innovative protection models and traditional approaches are necessary to endow traditional culture with new vitality and value[2].

The practical application of Intangible Cultural Heritage in design innovation mostly remains at the stage of aesthetic form, lacking systematic innovation from the perspective of cultural elements and craftsmanship [3]. Current shortcomings in design innovation for Intangible Cultural Heritage protection are mainly reflected in the following aspects: (1) innovation only in form, without a profound understanding of the importance of cultural values[4]; (2) excessive pursuit of marketization and commercialization, leading to homogenization of existing products. While commercialization can expand the scope of dissemination, the single-minded pursuit of economic benefits weakens the original cultural and historical significance of Intangible Cultural Heritage [5]; (3) limitations of design innovation methods. The failure to adopt modern design thinking and tools leads to creative exhaustion[6]; (4) the limitations of dissemination media affect the range and acceptance of Intangible Cultural Heritage [7]。. In conclusion, to address the limitations of Intangible Cultural Heritage protection methods and dissemination channels, researchers should explore innovative thinking and methods from the root and apply them to specific cases for refinement and iteration. In the field of design, updating and applying design methods is the source of innovation. **（Line32-56）**

**1-2.** Research objectives

The primary objective of this research is to explore the feasibility and innovativeness of generative design in the protection and inheritance of Intangible Cultural Heritage. By employing literature review and experimental methods, this study investigates the advantages of generative design in terms of the quantity and quality of design proposals. From the perspective of design thinking, three derivative product design models are proposed, further enriching the methods of Intangible Cultural Heritage protection and pathways for its inheritance. **（Line151-156）**

**1-3.** Innovation of this study

The innovations of this research are as follows: (1) Construction of a systematic design method. A new model for derivative product design is proposed, utilizing a method capable of generating diverse product design proposals in a short time; (2) Exhibition of diversified inheritance pathways. Various design carriers and methods are explored, including product design, digital design, and cultural innovation, enriching the design techniques and forms of Zhangzhou woodblock New Year picture derivatives; (3) Balancing innovative design with traditional inheritance. Through generative design, the works retain the characteristics of intangible cultural heritage while meeting modern aesthetic and practical needs. In summary, this research, from an industrial design perspective, explores the extraction and analysis of traditional elements and the integration of modern design methods to achieve a balance between traditional culture and contemporary design. **（Line157-167）**

**2.** Response to comment: Provides a simpler definition of generative design.

**Response:** Thank you for the professional guidance from the reviewer. After team discussion, this study has decided to replace the proprietary term "Derivative Design" with the broader term "Generative Design."

Generative design, originally stemming from the field of computer science, is a design methodology that generates a multitude of design options based on predetermined rules and optimizes and filters them according to specific conditions[24]. In industrial design, it typically refers to the method where designers use modern design tools to extract traditional design ele-ments and perform iterative design, systematic innovation, and derivative creation [25].

Generative design is widely applied in commercial design, engineering manufacturing, and the healthcare sector, characterized by its innovation, efficiency, low cost, automation, and scalability[26]. For instance, in product design, it assists designers in achieving intelligent de-sign processes, enhancing the innovation and competitiveness of derivative products [27]. In engineering manufacturing, it accelerates product iteration and diversifies derivative prod-ucts[28]; in architectural design, it provides new workflows and innovative methods for Building Information Modelling (BIM) [29]. In summary, generative design is an innovation-driven model based on rules, allowing designers to rapidly generate derivatives in various styles and categories. It can refresh the protection pathways of intangible cultural heritage and enrich the carriers of heritage transmission, possessing significant theoretical and practical value[30]**（Line336-350）**

**3.** Response to comment: Discuss the significance of derivative design and its relationship with intangible heritage. It is advisable to start from this point and then proceed to background information on Chinese woodblock New Year pictures, making it easier for readers to understand the importance of this study.

**Response:**

**3-1.** The significance of generative design

Generative design is a rule-driven innovation model widely used in fields such as architectural design, industrial design, engineering manufacturing, and commercial fashion. For in-stance, in industrial design, generative design can generate derivative product designs with different styles and aesthetic effects. Overall, generative design utilizes modern design tools to help designers break through traditional protection and transmission modes of intangible cultural heritage, exploring more diverse derivative design methods to achieve more efficient and innovative protection models [31]. **（Line351-357）**

**3-2.** Generative Design in Intangible Cultural Heritage Preservation

Generative design facilitates the digital protection and diversified transmission of intangible cultural heritage, with advantages in the following aspects:

（1）Digital Protection and Transmission: Generative design first digitizes traditional elements of intangible cultural heritage, such as patterns, characteristic designs, and craftsmanship processes. Then, using algorithms or design rules, it generates numerous innovative design proposals based on these traditional elements, preserving and transmitting traditional culture in new forms.

（2）Efficiency and Low Cost: As an innovation model based on elements or rules, generative design can produce a large number of design proposals in a short time, reducing protection costs and enriching transmission carriers.

（3）Balance Between Inheritance and Innovation: Generative design retains cultural characteristics through key information extraction while satisfying modern cultural and economic needs with diverse derivative design proposals[32].

In summary, generative design addresses the challenges in traditional intangible cultural heritage protection, such as conservative cultural elements, outdated functions, and misalignment with modern consumer habits, by providing diversified transmission pathways and ensuring the continuity of cultural value.

By extracting traditional patterns, color compositions, craftsmanship, and cultural elements of Chinese woodblock New Year pictures, generative design facilitates innovative derivative product designs that meet the demands for personalization, diversity, and rapid iteration in the AI era. Its advantages are evident in the following aspects:

（1）Redesign of Traditional Culture: Deconstructing and reconstructing traditional elements to establish innovative principles, leading to diverse design proposals.

（2）Digital Dissemination and Protection: Using generative design to digitally archive Zhangzhou woodblock New Year pictures and generate various digital works, enhancing the spread and efficiency of intangible cultural heritage.

（3）Commercialization and Industrialization: Generative design offers rich traditional carriers and pathways, which, when combined with the cultural tourism industry, can enhance regional economy and influence. **（Line359-387）**

In conclusion, intangible cultural heritage urgently needs more systematic protection methods, richer product carriers, and diversified transmission pathways to meet the needs of the times. **（Line407-408）**

**3-3.** Generative Design in woodblock New Year pictures Preservation

Generative design's deconstruction and reconstruction of Zhangzhou woodblock New Year pictures' design elements not only enriches protection modes and transmission pathways but also imbues them with new vitality and value, aligning them with the economic and cultural demands of modern society[33]. **（Line410-414）**

Special thanks to you for your good comments.

We tried our best to improve the manuscript and made some changes in the manuscript. These changes will not influence the content and framework of the paper. And here we did not list the changes but marked in red in revised paper.

We appreciate for Editors/Reviewers’warm work earnestly, and hope that the correction will meet with approval.

Once again, **Whether this article is published or not will determine whether my PhD will be postponed, so thank you again for your suggestions and help in my growth.**

**My sincere thanks again!!**
