# Supplementary material for: Revitalizing Intangible cultural heritage via derivative design: A focus on chinese woodblock printing
Source: PLoS One. 2025 Apr 17;20(4):e0318807. doi: 10.1371/journal.pone.0318807 (PMC12005565; doi:10.1371/journal.pone.0318807)
Supplement: Attachment — Submitted filename: Respond Reviewer#4 .docx [file pone.0318807.s005.docx]

Response to Reviewer # 4 （4 Comments）

**1. Summary**

Thank you very much for taking the time to review this manuscript. You are the strictest reviewer among all, and at the same time, the most important mentor in helping me elevate the quality of this research. Your encouragement is incredibly important to me and has boosted my confidence in academic research. I will dedicate more effort to enhancing my scholarly abilities in the future! In this revision, I added three references and a table, refined the introduction, and adjusted the numbering of the tables and figures accordingly. Thank you once again for your support on my academic journey!

**Comments 1:** a) Since most of readers are not familiar with the chinese woodblock printing, it would be better to add a few figures of this kind of printings.

Response 1:Dear Reviewer, Thank you for your professional advice, which has enriched the background of my research and enhanced the completeness of the experimental design. In response, I have added three references along with corresponding images. The specific revisions are as follows:

To further showcase the history, craftsmanship, cultural elements, and design features of Zhangzhou Woodblock New Year Pictures, this paper presents the cultural characteristics and historical evolution of woodblock prints through text, images, and videos. First, relevant information about its history and inheritance was extracted from the "China Intangible Cultural Heritage Network." It belongs to the category of traditional arts and crafts, with themes often covering historical stories, decorative patterns, and elements that convey wishes for happiness. Secondly, a video tutorial was used to demonstrate the craftsmanship and production process of Zhangzhou Woodblock New Year Pictures. Finally, three representative works were selected for cultural and semantic interpretation. The following discussion, from the perspectives of "blessing," "warding off evil spirits," and "folk customs," highlights these cultural and artistic features through the selected works. The specific representative works and descriptions of their cultural characteristics are shown in **Table 3**.***（line552-567）***

Table 3. Analysis of Cultural Characteristics of "Chinese Woodblock New Year Pictures" with Different Themes

| Representative Works |  |  | |  |
| --- | --- | --- | --- | --- |
| Name | Four Divine Beasts | The two spirits guarding the entrance to the house | The Mouse Wedding | |
| Cultural Implications | Auspicious designs symbolizing the pursuit of happiness. Their creative elements are drawn from classic stories, historical culture, or words of blessing. | Designs for warding off evil. Their creative elements are derived from historical figures or religious symbols. | | Decorative paintings showcasing local customs. Their creative elements stem from regional cultural characteristics and festive life scenes. |

**Comments 2:** b) The term "lion title sword" might means "a sword is holding by a lion head or a sword is bited by a lion" ("shi xian jian" in chinese), thus I suggest you to rethink the term and modify it.

**Response 2:** Thank you for your professional advice! Due to the limited research on "Chinese Woodblock New Year Pictures" in English, particularly regarding the regional "Zhangzhou Woodblock New Year Pictures," there is a lack of relevant terminology. In this paper, I have attempted to summarize its design features using straightforward descriptive language. Ultimately, I replaced "lion title sword" with "Lion Holding a Sword in Its Mouth," hoping that this simple translation can help readers more easily understand the characteristics of the design. Once again, thank you for your valuable suggestions!

**Comments 3:** c) Line 70, you mentioned that the "traditinal themes cannot meet the spiritual needs of users in the new era", however, I cannot agree with you because some traditional stories or themes are still accepted by modern people partly.

**Response 3:** Your suggestions are excellent! They further broaden the research perspective of this paper, allowing both tradition and modernity to be understood and passed down through time. As a result, I have revised this section as follows:

Traditional intangible cultural heritage still retains its vitality, conveying national culture and historical symbols. Its craftsmanship showcases how technology can drive cultural dissemination, while its cultural traits reflect regional customs, fostering intercultural exchange and historical inheritance. However, with the advancement of technology and the impact of modern culture, traditional woodblock prints face challenges such as limited mediums of dissemination, gaps in transmission paths, and insufficient commercial development. Therefore, it is urgently necessary to utilize derivative design to provide more diverse dissemination platforms, wider channels of communication, and approaches that better align with contemporary societal needs. ***（line70-77）***

**Comments 4:** d) Additonally, the numbers of figues and table should be clearly matched to the text.

**Response 4:** Thank you very much for your reminder. Your rigorous academic attitude has set a great example for me. I have revised the numbering and quantity of the figures and tables accordingly. Additionally, a new table has been added, and the sequence has been adjusted. Once again, thank you for your valuable suggestions!

I would like to express my gratitude once again! I spent a week thoroughly reading your suggestions, which have been immensely beneficial and provided me with invaluable assistance!

Thank you for your meticulous guidance and assistance. Your suggestions have been invaluable to me!
