# Supplementary material for: Revitalizing Intangible cultural heritage via derivative design: A focus on chinese woodblock printing
Source: PLoS One. 2025 Apr 17;20(4):e0318807. doi: 10.1371/journal.pone.0318807 (PMC12005565; doi:10.1371/journal.pone.0318807)
Supplement: Attachment — Submitted filename: Response to Reviewer #8 .docx [file pone.0318807.s006.docx]

Response to Reviewer #8’s Comment

Thank you very much for taking the time to review this manuscript.

Your encouragement is very important to me and has strengthened my confidence in academic research.

I will work harder to improve my academic ability in the future!

Thank you again and I wish you a happy life! ^_^
